# Supplementary material for: ATP-Triggered Conformational Changes Delineate Substrate-Binding and -Folding Mechanics of the GroEL Chaperonin
Source: Cell. 2012 Mar 30;149(1):113–23. doi: 10.1016/j.cell.2012.02.047 (PMC3326522; doi:10.1016/j.cell.2012.02.047)
Supplement: Document S1. Tables S1 and S2 [file mmc1.pdf]

Table S1. Domain rotations and translations, related to Figures 4 and 5.

A.

Singly-bound top ring

GroEL-ATP$_7$ conformations		Translation (Å)	Rotation (degrees)	Rotation axis x	y	z
**Apical domains**						
Proposed trajectory						
Apo	Rs1	13.8	36.9	0.4	-0.6	0.7
Rs1	Rs2	3.0	8.9	1.0	0.1	-0.2
Rs2	Rs-open	10.3	17.7	0.1	0.4	-0.9
Rs-open	R-ES	7.6	95.6	-0.5	0.9	-0.2
**Intermediate domains**						
Proposed trajectory						
Apo	Rs1	11.7	35.3	0.4	-0.6	0.7
Rs1	Rs2	1.8	2.7	-0.8	0.5	-0.4
Rs2	Rs-open	1.7	6.3	-0.2	-0.8	0.5
Rs-open	R-ES	4.5	14.6	0.8	0.6	-0.3
**Equatorial domains**						
Proposed trajectory						
Apo	Rs1	2.0	8.4	1.0	0.1	0.1
Rs1	Rs2	0.8	2.8	-1.0	0.0	-0.1
Rs2	Rs-open	1.6	2.8	0.3	-1.0	-0.1
Rs-open	R-ES	5.2	7.4	0.0	0.8	-0.6

B.

Double-bound top ring

GroEL-ATP$_{14}$ conformations	Translation (Å)	Rotation (degrees)	Rotation axis x	y	z
**Apical domains**					
Proposed trajectory					

Apo	Rd1	8.8	40.8	-0.4	-0.6	-0.7
Rd1	Rd2	5.1	8.0	0.6	-0.4	0.7
Rd2	Rd-open	13.7	24.4	-0.7	-0.7	-0.2
Rd-open	R-ES	6.9	107.6	-0.1	0.5	0.8
**Intermediate domains**						
Proposed trajectory						
Apo	Rd1	6.7	36.5	-0.3	-0.6	-0.7
Rd1	Rd2	1.8	3.4	0.9	-0.2	-0.4
Rd2	Rd-open	1.7	2.6	-0.2	0.5	0.9
Rd-open	R-ES	2.0	11.4	0.7	-0.1	0.7
**Equatorial domains**						
Proposed trajectory						
Apo	Rd1	0.5	6.4	0.5	0.8	-0.3
Rd1	Rd2	0.8	2.1	0.2	-0.5	0.9
Rd2	Rd-open	1.0	4.7	0.0	-0.3	-1.0
Rd-open	R-ES	0.4	5.2	-0.3	0.5	0.8
**Double-bound bottom ring**						
**Apical domains**						
Proposed trajectory						
Apo	Rd3	7.3	29.3	0.3	-0.6	0.7
Rd3	Rd4	3.7	11.2	0.4	-0.8	0.3
Rd4	Rd5	6.9	7.8	-0.7	-0.5	-0.5
Rd5	R-ES	7.9	95.7	0.4	0.6	-0.7
**Intermediate domains**						
Proposed trajectory						
Apo	Rd3	5.8	29.4	0.3	-0.6	0.7
Rd3	Rd4	3.4	11.3	0.1	-0.5	0.9
Rd4	Rd5	4.2	10.9	-0.2	-0.2	-0.9
Rd5	R-ES	4.5	7.0	-0.7	0.5	-0.4
**Equatorial domains**						
Proposed trajectory						
Apo	Rd3	0.1	0.5	-0.2	0.9	0.2

Rd3	Rd4	1.0	4.5	0.1	-0.9	0.4
Rd4	Rd5	0.8	8.8	-0.4	-0.7	0.5
Rd5	R-ES	1.2	13.6	0.2	0.9	-0.4

Table S2. Intersubunit contact distances (Å), related to Figures 4 and 5.

A.

Singly-bound top ring	GroEL-ATP$_7$ conformation				
**Residues**	**T**	**RS$_1$**	**RS$_2$**	**RS-open**	**R-ES**
**inter subunit**					
Arg197-Cζ Glu386-Cδ	4.6	22.9	23.5	32.1	34.8
Lys80–Nζ Glu386-Cδ	17.8	4.0	5.3	11.0	7.9
Glu255-Cδ Lys207-Nζ	4.0	17.5	16.5	32.8	40.6
Glu255-Cδ Lys245-Nζ	16.4	2.9	3.1	22.6	47.5
**inter ring**					
Glu461-Cδ Arg452-Cζ	5.2	4.7	4.6	6.0	4.4
Ala109-Cβ Ala109-Cβ	3.6	6.6	6.4	6.6	7.3

B.

Double-bound rings	GroEL-ATP$_{14}$ conformation							
**Residues**	**T**	**RD$_1$**	**RD$_2$**	**RD$_3$**	**RD$_4$**	**RD$_5$**	**RD-open**	**R-ES**
**inter subunit**								
Arg197-Cζ Glu386-Cδ	4.6	19.3	26.4	26.9	25.6	28.0	35.6	34.8
Lys80-Nζ Glu386-Cδ	17.8	11.8	12.3	9.8	9.7	13.2	11.3	7.9
Glu255-Cδ Lys207-Nζ	4.0	16.2	18.5	21.9	20.4	22.6	37.9	40.6
Glu255-Cδ Lys245-Nζ	16.4	6.5	3.8	9.1	7.7	3.3	26.4	47.5
Glu257-Cδ Lys245-Nζ	17.5	8.4	3.4	3.0	3.1	7.0	27.4	51.8
Glu257-Cδ Lys242-Nζ	21.5	18.6	15.7	14.6	14.9	3.0	21.2	44.9
**inter ring**								
Glu461-Cδ Arg452-Cζ	5.2	5.9	4.0	5.9	4.0	4.6	4.6	4.4
Ala109-Cβ Ala109-Cβ	3.6	6.8	7.8	6.8	7.8	8.6	8.6	7.3
