# Supplementary figures and images for: ATP-Triggered Conformational Changes Delineate Substrate-Binding and -Folding Mechanics of the GroEL Chaperonin
Source: Cell. 2012 Mar 30;149(1):113–23. doi: 10.1016/j.cell.2012.02.047 (PMC3326522; doi:10.1016/j.cell.2012.02.047)

## Movie S1

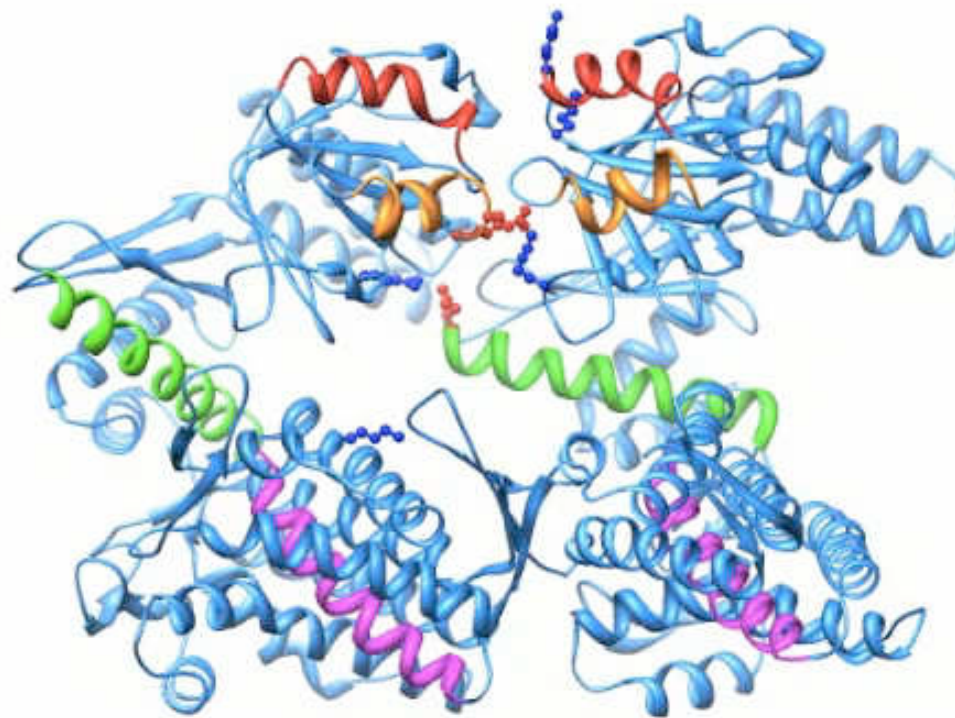

# Movie S2

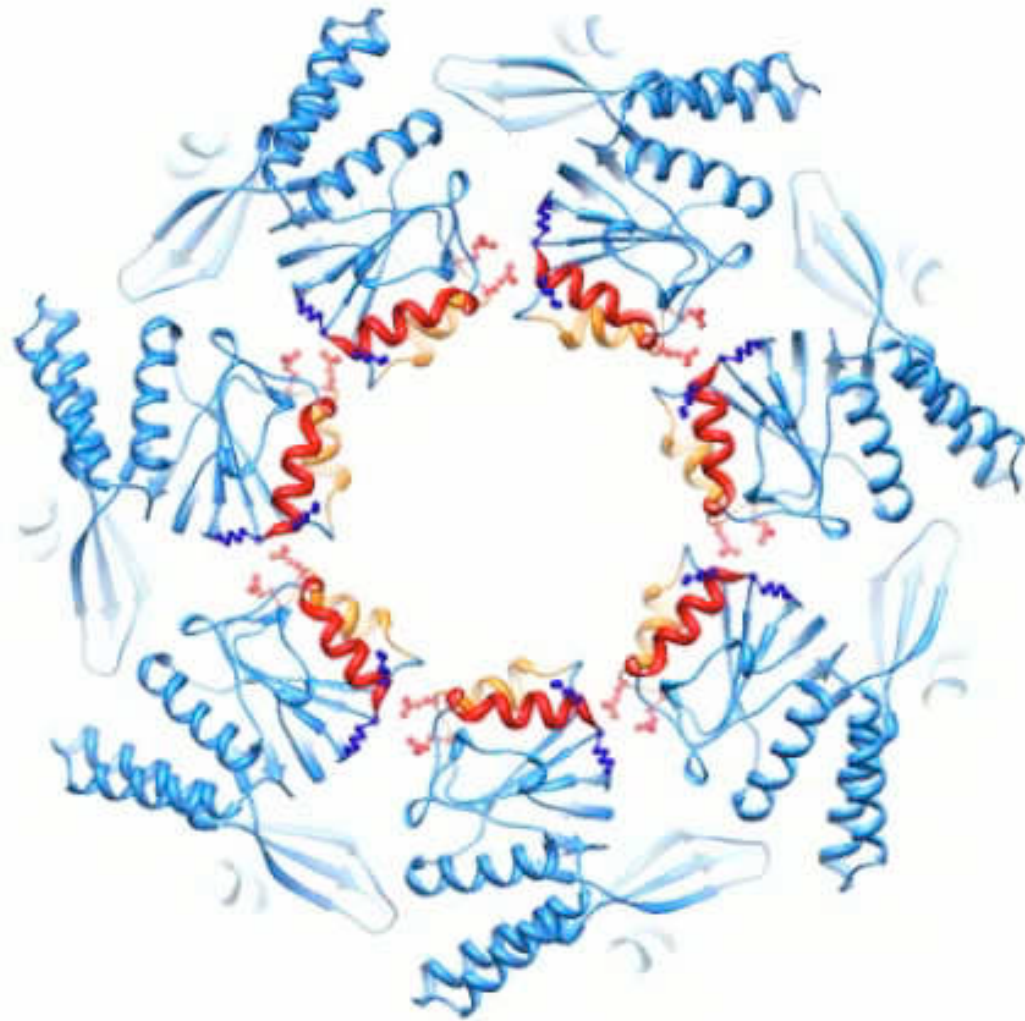

Supplement: Document S2. Movies S1 and S2, Related to Figure 4 — Note: These two movies are PDF files that should be viewed with Adobe Acrobat Reader software (and not Preview, for Mac users). Movie S1: Morph showing the domain movements and salt-bridge switching for two subunits of GroEL-ATP7. The morph was created by interpolating between T, Rs1, Rs2 Rs-open, and R-ES conformations. The view is from the inside of the ring. The morphs shown in Movies S1–S4 were made with UCSF Chimera (Pettersen et al., 2004). Movie S2: Morph of GroEL-ATP7 ring viewed from above, showing the tilts and rotations of the apical domains. [file mmc2.pdf]

## Movie S3

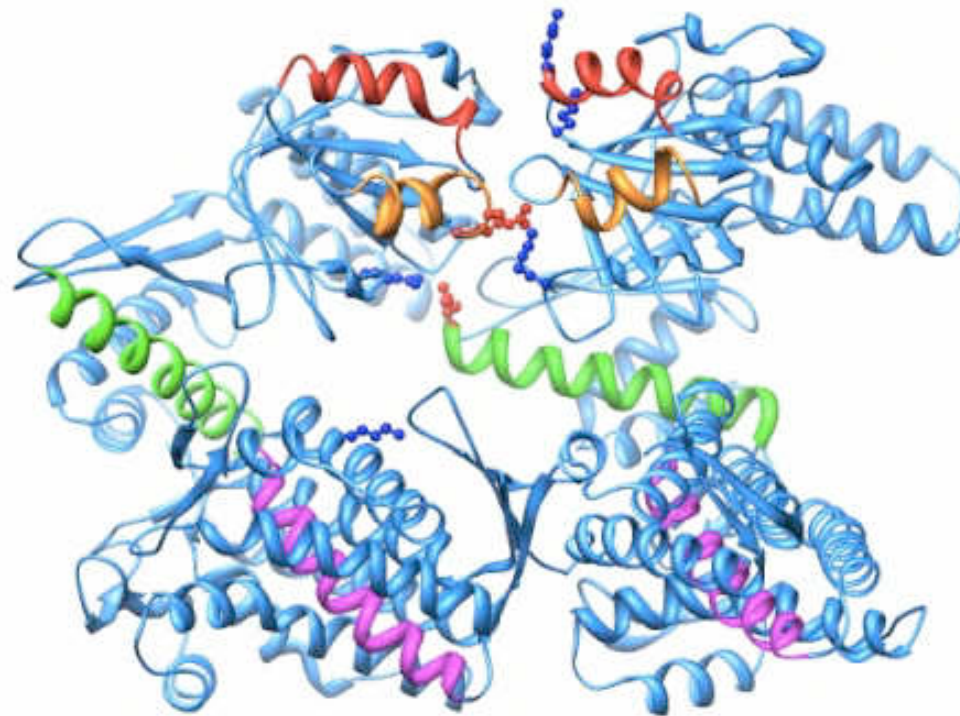

# Movie S4

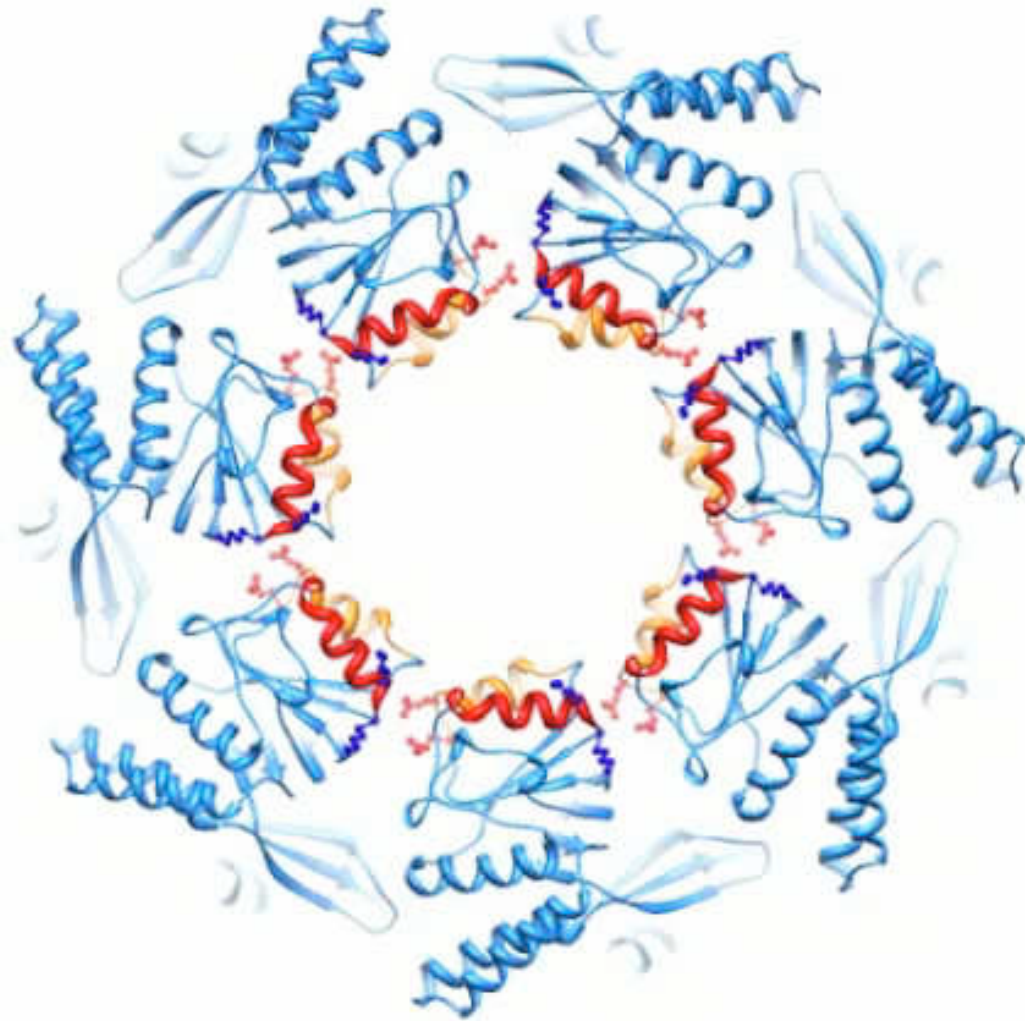

Supplement: Document S3. Movies S3 and S4, Related to Figure 4 — Note: These two movies are PDF files that should be viewed with Adobe Acrobat Reader software (and not Preview, for Mac users). Movie S3: Morph showing the domain movements and salt-bridge switching for two subunits of GroEL-ATP14 Morph of two subunits as in Movie S1, but for T, Rd1, Rd2, Rd3, Rd5, Rd-open, and R-ES conformations. Movie S4: Ring view of the GroEL-ATP14 morph in Movie S3. [file mmc3.pdf]

## Movie S5

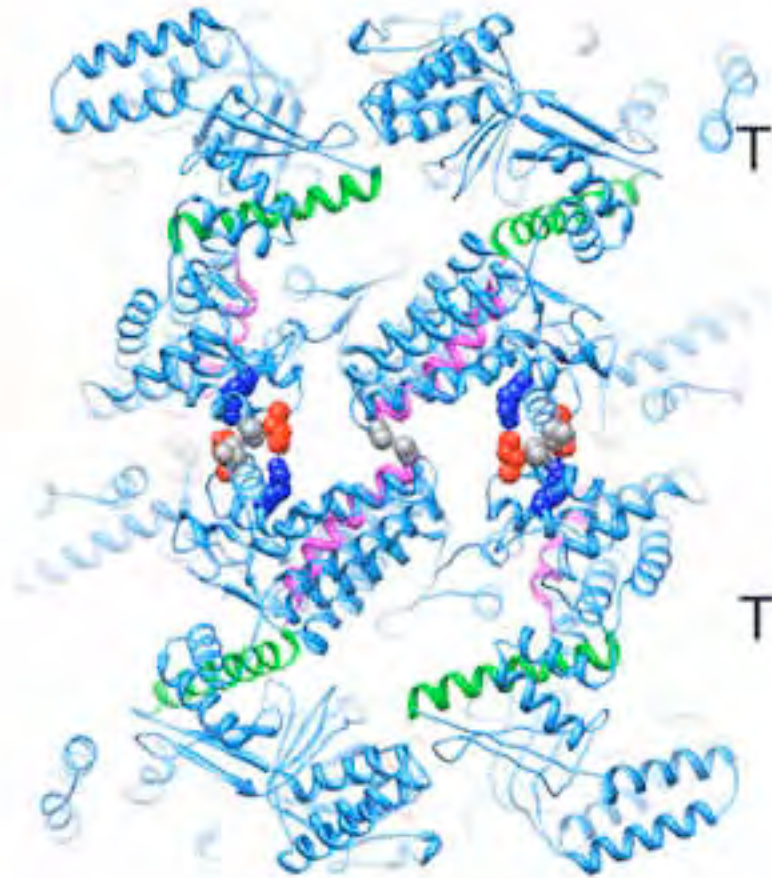

## Movie S6

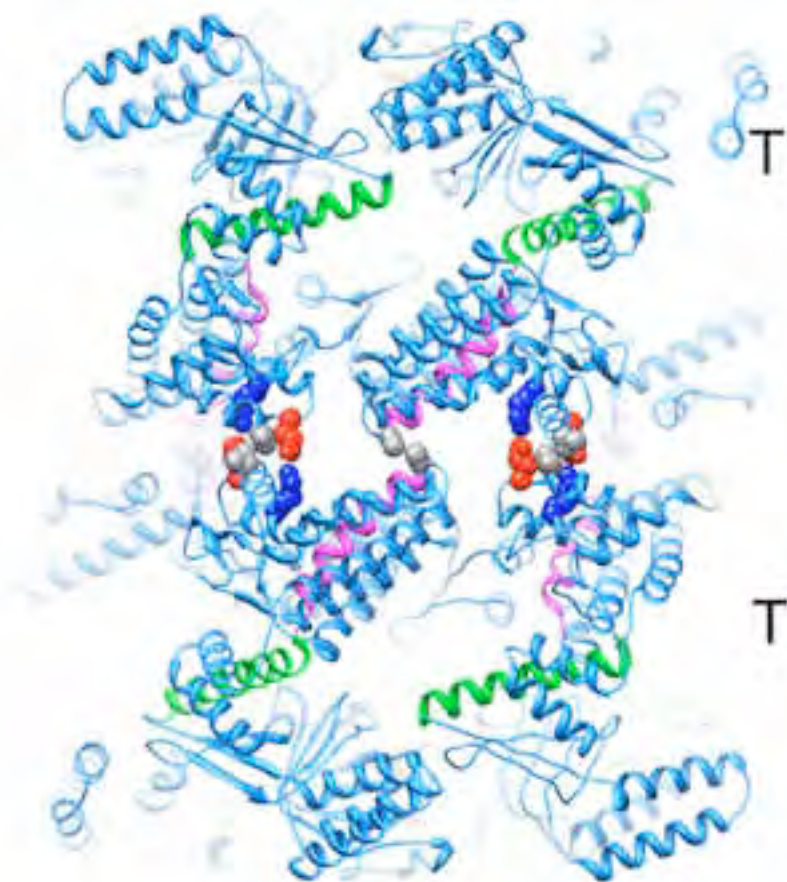

## Movie S7

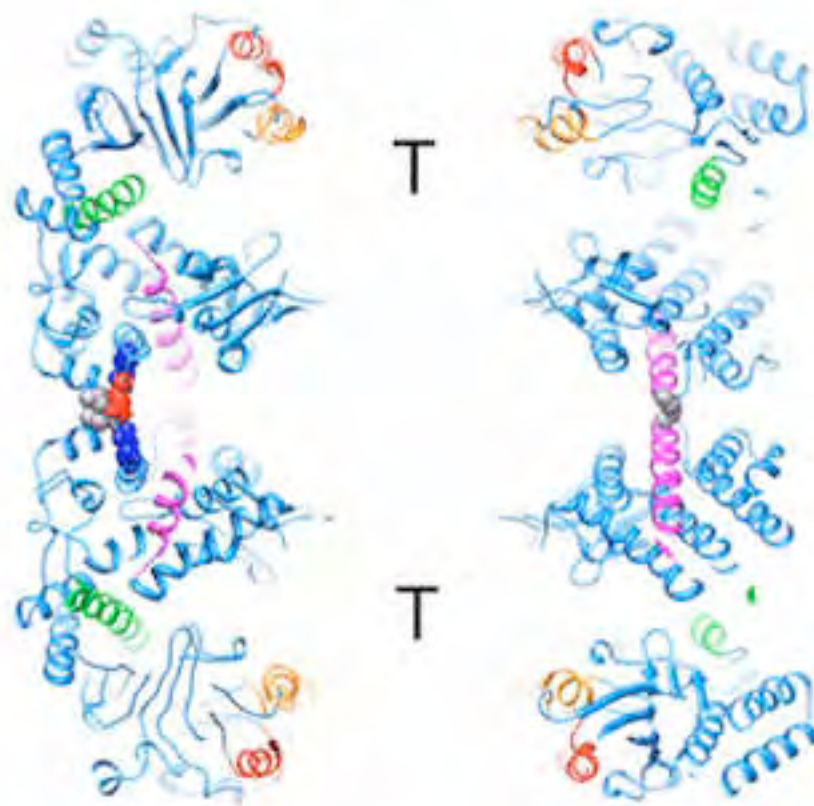

## Movie S8

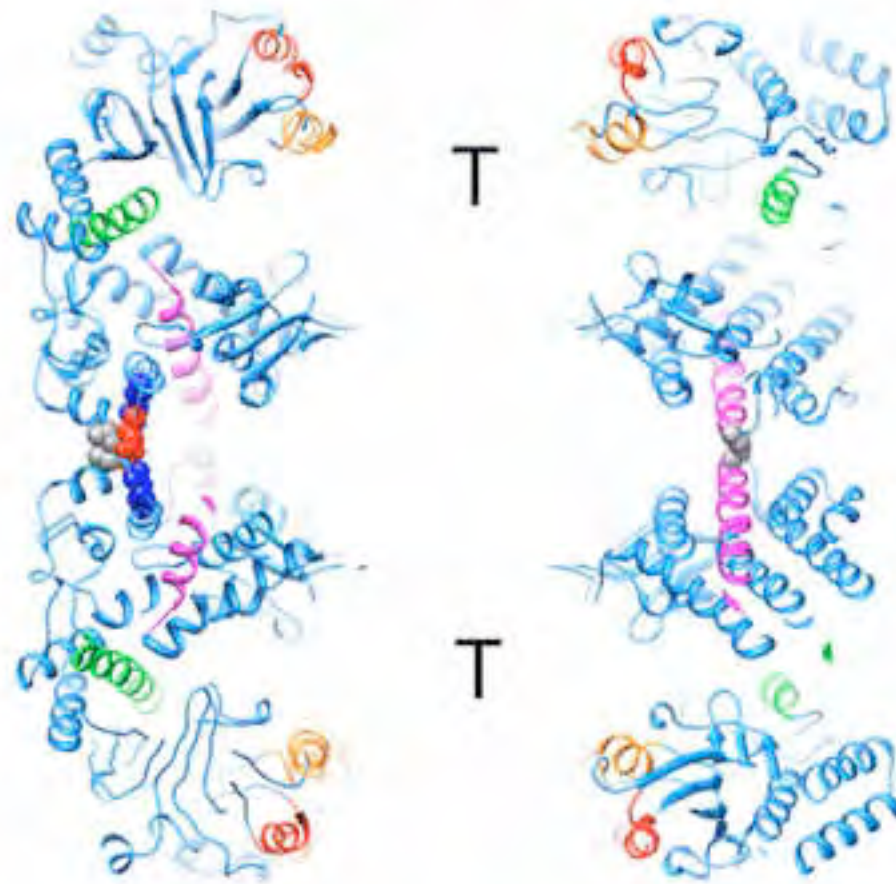

Supplement: Document S4. Movies S5–S8, Related to Figure 5 — Note: These four movies are PDF files that should be viewed with Adobe Acrobat Reader software (and not Preview, for Mac users). Movie S5: Flip movie of a front view of GroEL-ATP7. The frames alternate between T:T, Rs1:T, Rs-open:T, and R-ES:T states, seen from outside the complex. The largest movements are in the apical domains, but equatorial domain tilts also alter the inter-ring contacts. The A109 contact is seen in the center, flanked by two E461/R452 contacts. Side chains of these contact residues are shown in space-filling format. The en bloc, 35° tilt of intermediate and apical domains can be seen in the step from T:T to Rs1:T states. Movie S6: Flip movie of GroEL-ATP14 states displayed as in Movie S5. The frames alternate between T:T, Rs1:T, and Rd-open:Rd5 complexes to show the range of domain movements and expansion of the inter-ring interface as ATP binds first to one ring and then to both. Movie S7: Flip movie of GroEL-ATP7 states seen as central slices through the complex. The frames alternate between T:T, Rs1:T, Rs2:T, Rs-open:T, and R-ES:T states. Side chains of the contact residues (E461 and R452 on the left and A109 on the right) are shown in space-filling format. Movie S8: Flip movie of GroEL-ATP14 states seen as central slices through the complex. The frames alternate between T:T, Rs1:T, Rs2:T, and Rd-open:Rd5 states. [file mmc4.pdf]
