# Supplementary material for: ATP-Triggered Conformational Changes Delineate Substrate-Binding and -Folding Mechanics of the GroEL Chaperonin
Source: Cell. 2012 Mar 30;149(1):113–23. doi: 10.1016/j.cell.2012.02.047 (PMC3326522; doi:10.1016/j.cell.2012.02.047)
Supplement: Document S5. Article plus Supplemental Information [file mmc5.pdf]

ATP-Triggered Conformational Changes Delineate Substrate-Binding and -Folding Mechanics of the GroEL Chaperonin

Daniel K. Clare,[1] Daven Vasishtan,[1] Scott Stagg,[2,6] Joel Quispe,[2] George W. Farr,[4,5,7] Maya Topf,[1] Arthur L. Horwich,[3,4,5] and Helen R. Saibil[1,*]
[1]Crystallography and Institute of Structural and Molecular Biology, Birkbeck College, University of London, Malet Street, London WC1E 7HX, UK
[2]The National Resource for Automated Molecular Microscopy, Department of Cell Biology
[3]Department of Molecular Biology
The Scripps Research Institute, 10550 North Torrey Pines Road, La Jolla, CA 92037, USA
[4]Department of Genetics
[5]Howard Hughes Medical Institute
Yale University School of Medicine, Boyer Center, 295 Congress Avenue, New Haven, CT 06510, USA
[6]Present address: Department of Chemistry and Biochemistry, Institute of Molecular Biophysics, Florida State University, 91 Chieftan Way, Tallahassee, FL 32306-4380, USA
[7]Present address: Aeromics LLC, 11000 Cedar Avenue, Cleveland, OH 44106-3069, USA
*Correspondence: h.saibil@mail.cryst.bbk.ac.uk
DOI 10.1016/j.cell.2012.02.047

SUMMARY

The chaperonin GroEL assists the folding of nascent or stress-denatured polypeptides by actions of binding and encapsulation. ATP binding initiates a series of conformational changes triggering the association of the cochaperonin GroES, followed by further large movements that eject the substrate polypeptide from hydrophobic binding sites into a GroES-capped, hydrophilic folding chamber. We used cryo-electron microscopy, statistical analysis, and flexible fitting to resolve a set of distinct GroEL-ATP conformations that can be ordered into a trajectory of domain rotation and elevation. The initial conformations are likely to be the ones that capture polypeptide substrate. Then the binding domains extend radially to separate from each other but maintain their binding surfaces facing the cavity, potentially exerting mechanical force upon kinetically trapped, misfolded substrates. The extended conformation also provides a potential docking site for GroES, to trigger the final, 100° domain rotation constituting the "power stroke" that ejects substrate into the folding chamber.

INTRODUCTION

Chaperonins are a major class of molecular machines that provide assistance to protein folding in cells. They enable the folding of a diverse subset of cellular proteins by a nonspecific mechanical action involving coordinated, large excursions of the chaperonin domains in a double-ring assembly. Currently best understood is the bacterial chaperonin GroEL, which assists the folding of nascent or stress-denatured polypeptides in steps of binding and encapsulation (Figure 1) (Thirumalai and Lorimer, 2001; Hartl and Hayer-Hartl, 2002; Horwich and Fenton, 2009). The non-native polypeptide is first captured on a ring of hydrophobic sites lining cavities at the ends of the barrel-shaped complex. Then, the combined actions of ATP and the cochaperonin GroES trigger a dramatic series of concerted domain rotations that eject the polypeptide from the cavity wall and encapsulate it for folding in isolation, inside a hydrophilic chamber capped by GroES (Mayhew et al., 1996; Weissman et al., 1996; Xu et al., 1997).

The key steps in this structural transition from an open hydrophobic ring to an enclosed folding chamber have not been determined, although computational methods have been used with the aim of predicting the trajectory of these movements (Ma et al., 2000; Tehver et al., 2009; Yang et al., 2009). The process is initiated by concerted ATP binding to a GroEL ring, rapidly followed by initial contact with GroES, and concludes with additional rigid-body movements of the GroEL apical domain to form the GroES-domed chamber. ATP binds with positive cooperativity within rings but with negative cooperativity between rings (Yifrach and Horovitz, 1995) and is followed by substrate binding to the hydrophobic sites (Tyagi et al., 2009). Once the substrate is encapsulated in the domed chamber, the slow hydrolysis of ATP (half-time ~10 s) provides a time window for folding to take place, but ATP hydrolysis is not required for protein folding. Rather, hydrolysis moves the machine forward through its reaction cycle. Hydrolysis in the GroES-bound ring is a prerequisite for ATP binding to the opposite ring, which in turn triggers the allosteric discharge of GroES, ADP, and substrate, whether folded or not, from the folding chamber. If the released substrate is not correctly folded, it can rebind for further cycles of interaction with the chaperonin (Rye et al., 1999).

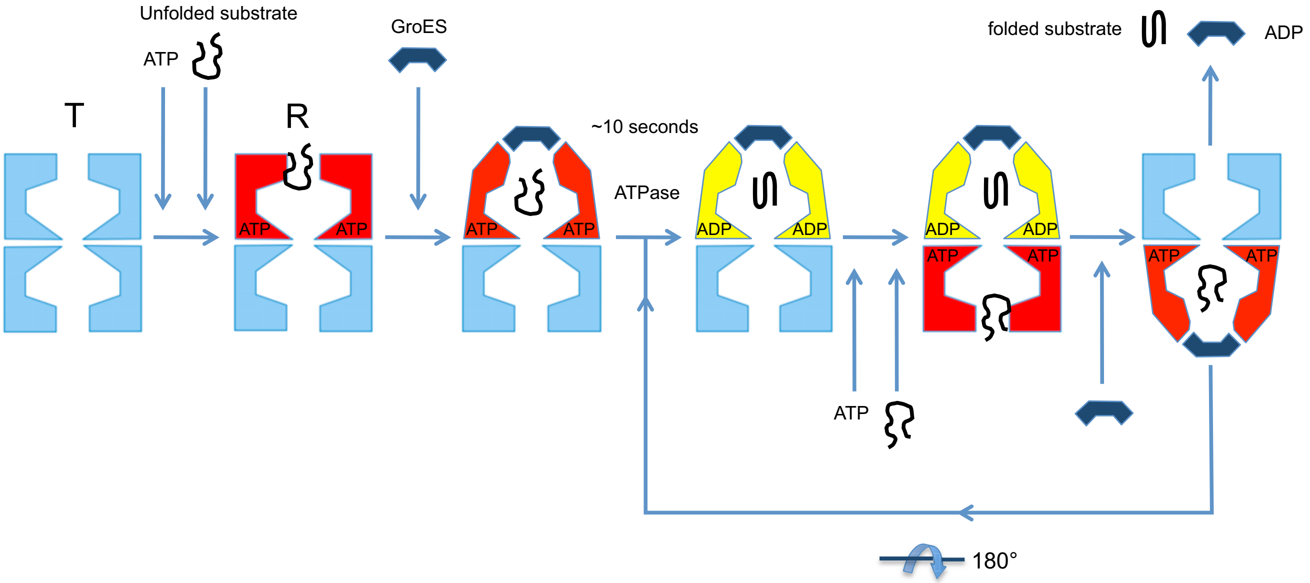

Figure 1. Diagram of the GroEL-GroES ATPase Cycle, Showing the Steps of Substrate Binding, Encapsulation, Folding, and Release
ATP hydrolysis in one ring is required to enable subsequent ATP binding to the opposite ring, but hydrolysis is not required for folding to proceed within the chamber.

Crystal structures of GroEL as well as cryo-electron microscopy (cryo-EM) studies show the two extremes of its trajectory of domain movements (Braig et al., 1994; Roseman et al., 1996; Xu et al., 1997). Comparison of the apo GroEL structure with the GroES-bound form revealed substantial rigid-body rotations about interdomain hinge points, in addition to minor local rearrangements of secondary structure elements within the domains (Xu et al., 1997). Cooperative ATP binding to a unique pocket in the equatorial domains induces a downward rotation of the intermediate domains (Ranson et al., 2001; Ma et al., 2000). This rotation breaks the intersubunit, intermediate-to-apical domain contact E386-R197 that plays a key role in allostery (White et al., 1997) and replaces it with an intersubunit, E386-K80 intermediate-to-equatorial domain contact (Ranson et al., 2001). In the presence of nucleotide and GroES, a major elevation and twist of the apical domains (Roseman et al., 1996; Xu et al., 1997) move the hydrophobic polypeptide-binding site on each apical domain from a position facing the cavity in apo GroEL to an elevated and rotated position in which part of each hydrophobic site binds a mobile loop of the GroES heptamer to complete the domed structure of the folding chamber. Mutational mapping of the hydrophobic binding sites showed an overlap between substrate and GroES binding (Fenton et al., 1994). Competitive displacement of substrate by GroES could account for its simultaneous ejection from the binding surface and encapsulation in the folding chamber but does not explain why there is no escape of substrate into solution at this step.

The role of the ATP γ-phosphate is critical in substrate encapsulation (Motojima et al., 2004). In the absence of substrate, the structure of the folding chamber looks almost the same when formed with ADP as with ATP (Chaudhry et al., 2003; Ranson et al., 2006). However, ADP and GroES are not sufficient to eject stringent substrates from their binding sites on GroEL, whereas ATP and GroES trigger ejection and permit folding to proceed. Motojima et al. used FRET to report on apical domain movements in order to show that bound substrate introduces a load on the apical domains, slightly slowing their motion in ATP and both severely slowing their motion and preventing their full extension to the final GroES-bound state in ADP. Adding the γ-phosphate analog aluminum fluoride to the ADP complex releases this block of productive folding and triggers substrate refolding at the same rate as in ATP.

Observations of multiple GroEL-ATP fluorescent phases in earlier kinetic studies suggest that there are intermediate conformations on the trajectory from the apo to ATP/GroES-domed form (Cliff et al., 1999, 2006; Yifrach and Horovitz, 1998; Horovitz et al., 2002). This indicates that the earlier cryo-EM reconstruction of ATP-bound GroEL (Ranson et al., 2001) probably represented a mixture of states. Averaging multiple conformations would account for the missing density and limited resolution in that study and suggests that the earlier interpretation of ATP-induced movements based on docking of atomic structures is unlikely to be accurate in detail.

Here we have carried out statistical analysis of a large data set (60,000 images) of single-particle cryo-EM images of a GroEL mutant deficient in ATP hydrolysis in the presence of ATP. We resolve multiple pre-hydrolysis conformations that can be ordered into a sequence to trace out smooth trajectories of domain movements for GroEL-ATP_7 and GroEL-ATP_{14} complexes. The structures reveal a set of salt-bridge changes that provide a series of "click stops" (preferred conformations) on a trajectory to a conformation in which the apical domains are separated from each other and partially elevated but lack

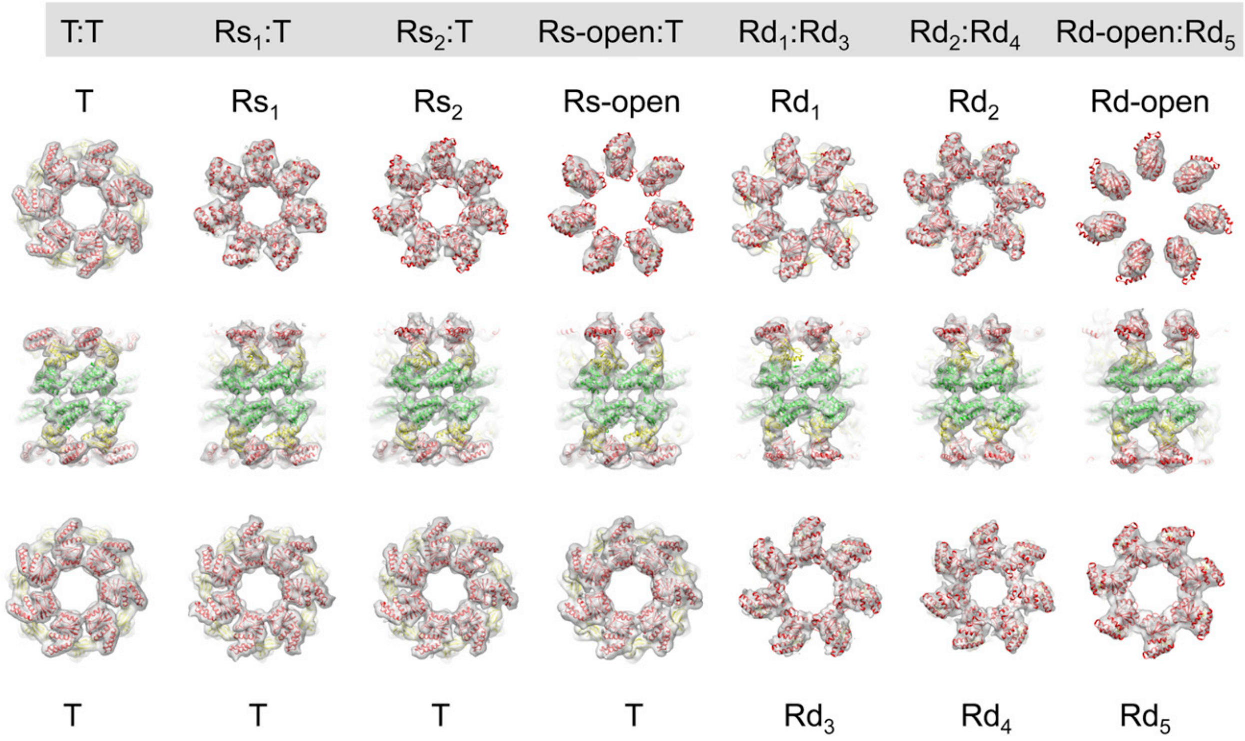

Figure 2. Cryo-EM Maps of the Seven Structures Determined from the GroEL-D398A-ATP Data Set

Maps are shown as white surfaces. Top ring, side view, and bottom ring are shown for each complex. The atomic structures are fitted into the maps, with the equatorial domain in green, intermediate in yellow, and apical in red. T, tense allosteric state (unliganded); R, relaxed states (ATP bound). Rs rings are the ATP-bound rings from GroEL-ATP_7 complexes (single), and Rd rings are from GroEL-ATP_{14} complexes (double). Figure produced with UCSF Chimera (Pettersen et al., 2004). See also Figures S1, S2, and S3.

the full elevation and large clockwise twist seen in the GroES-bound rings. This elevated, open conformation of the GroEL ring positions the GroES-binding sites on its apical surface, while still exposing key hydrophobic sites toward the cavity, suggesting how the chaperonin can recruit GroES before ejecting the substrate from its binding sites. It also suggests how the final large twist of the apical domains provides the power stroke to eject substrate from the hydrophobic sites.

RESULTS

Cryo-EM Maps and Atomic Structure Fitting of GroEL-ATP Complexes

In this study, we aimed to discriminate the multiple conformations induced upon ATP binding to GroEL. We used an ATPase mutant (D398A) with normal ATP binding but 3% of the wild-type steady-state ATPase activity. It is nonetheless able to efficiently encapsulate and fold proteins (Rye et al., 1997, 1999). The sample was briefly incubated with ATP and vitrified. Because the side views, which are essential for three-dimensional (3D) reconstruction, accounted for only 5%–10% of the total, we used the Leginon automated data collection system (Suloway et al., 2005) to obtain 6,000 CCD frames, yielding ~60,000 side views suitable for analysis.

We used multivariate statistical analysis (MSA; Elad et al., 2008; Orlova and Saibil, 2010) to progressively subdivide the particles on the basis of eigenimages distinguishing conformational changes in a ring from orientational variations (Figure S1 available online). The separated classes produced six distinct 3D reconstructions of GroEL-ATP states, three with ATP bound in one ring and three with ATP in both rings, in addition to an unliganded (apo GroEL) population (Figure 2). The map resolution was 7 Å for apo GroEL and 8–9 Å for GroEL-ATP, with ATP density visible in occupied nucleotide pockets and almost all helical secondary structures resolved (Figure 3).

In order to analyze the domain movements as accurately as possible, we used flexible fitting (Topf et al., 2008) to dock GroEL crystal structures into our maps followed by energy minimization to further optimize side-chain conformations (Figure S3) (Phillips et al., 2005). This procedure maintains the connectivity between the GroEL domains and allows deformation of specified hinge regions. With this approach, we were able to accurately model the conformation of all the complexes, and we adopt an elaboration of the T/R (tense/relaxed) terminology of allosteric states (Yifrach and Horovitz, 1995) to label the rings (Figure 2). ATP-bound rings from GroEL-ATP_7 complexes are termed Rs (single), and those from GroEL-ATP_{14} Rd (double). For the Rs states, the fitted models reveal three distinct new conformations between the T state (apo GroEL) and the GroES-bound state (R-ES), termed Rs1, Rs2, and Rs-open. The Rd states were identified as six different ring conformations in the following double-ring complexes: Rd1:Rd3, Rd2:Rd4, and Rd-open:Rd5. Each state

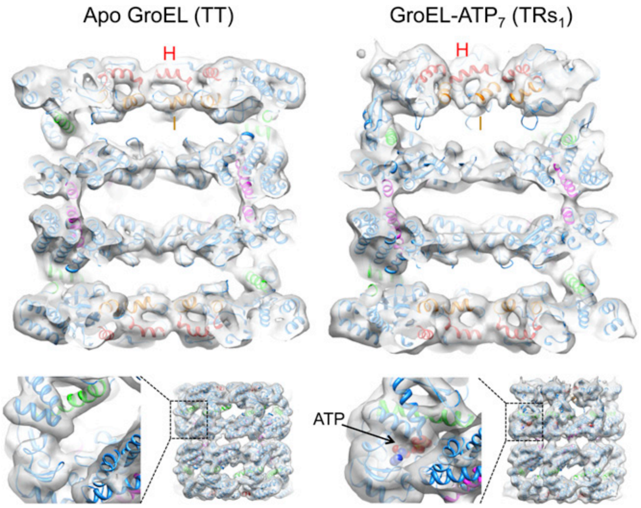

Figure 3. Cut-Open Views of the EM Maps and Fits of Apo GroEL and the GroEL-ATP$_7$ Complex with the Top Ring in the Rs1 State and the Bottom Ring in the T State

The EM density is in white, and apical domain helices H and I, defining the substrate-binding site, are in red and orange, respectively. Intermediate domain helix M (green) contains the catalytic aspartate that contacts the nucleotide, and equatorial domain helix D (magenta) runs from the γ-phosphate to an inter-ring contact. The fitting shows that α-helical secondary structures are largely resolved in these maps. Shown below each complex is a view of the region around the ATP-binding pocket, which is empty in the T state but filled with density in the Rs1 state. The ATP molecule inside the Rs1 density is shown as spheres with CPK coloring. See also Figure S3.

is defined by a set of intersubunit salt bridges, which are clearly seen as bridges of density. The structures are shown in the order corresponding to the smallest steps in domain rotation between successive conformations. For both sequences, the major movements are first a large, en bloc rotation of the intermediate and apical domains forming the Rs and Rd states, followed by elevation and radial expansion of the apical domains to the R-open states.

In the above analysis, we imposed 7-fold symmetry, in accordance with the known positive cooperativity of ATP binding within GroEL rings (Yifrach and Horovitz, 1995). However, we also explored asymmetric refinement of each structure to check for evidence of asymmetric movements. The only states in which significant asymmetry appeared are the critical steps of apical domain detachment leading to the R-open states. In particular, asymmetric refinement of the Rs2 state suggested that 1–2 apical domains detach first and begin to elevate before the others. However, almost all subunits in the R-open states appear to be elevated. In some of the states, the apical domain hairpins formed by helices K and L become disordered, as previously observed in the unliganded rings of GroEL-GroES-ADP and GroEL-GroES-ATP complexes (Ranson et al., 2006).

Domain Movements and Switching of Intersubunit Salt Bridges

The domain rotations and translations are listed in Table S1 and shown graphically for the apical domains in the major steps: apo to Rs1, Rs1 to Rs2, Rs2 to Rs-open, and Rs-open to R-ES (Figure S4). The movements in the ATP-bound rings produce obvious differences in intersubunit contacts (Figure 4). The sequence of breakage and formation of intersubunit contacts is consistent with the order of the Rs and Rd ring conformations chosen as explained above (Figures 2 and 4). The contact distances are plotted in Figure S5 and tabulated in Table S2.

Very similar patterns are seen in the Rs and Rd series, but the Rs series is simpler and is sufficient to illustrate the major steps in the trajectory. The initial step after ATP binding is an en bloc, 35° sideways tilt of the intermediate and apical domains as a single rigid body (to the left in Figure 4, top panel, Rs1; see Movie S5) about the equatorial-intermediate hinge. Pivoting of these domains about the equatorial-intermediate hinge closes helix M (green, containing the catalytic residue D398 in the native structure) over the ATP-binding pocket. The tilt breaks the salt bridge between intermediate and apical domains of adjacent subunits (R197-E386) in apo GroEL and replaces it by a new contact between K80 and E386 in the adjacent equatorial domain, as previously described (Ranson et al., 2001). In addition, the contact E255-K207 between adjacent apical domains of apo GroEL is broken and replaced by a contact between E255 at the end of helix I (orange) and K245 at the end of helix H (red) on the next subunit. In the next step (Rs1-Rs2), the intermediate-apical hinge also bends, so that the apical domain undergoes an additional elevation, with the two new salt bridges remaining connected (Rs2). Finally, both of these salt bridges are broken, and the apical domain moves radially outwards and elevates 20° to form the Rs-open state. Although the apical domain in the R-open state has completed about 70% of the final elevation observed in R-ES, the lack of twist means that helix I and the underlying segment, part of the substrate-binding site, are still accessible from the cavity. To reach the R-ES conformation, the apical domains must twist ~100° clockwise and complete their elevation (see Table S1). The movements can be followed in Movie S1, which shows the succession of apical domain tilt, elevation and twisting movements, along with smaller rocking and tilting motions of the equatorial domains. The twisting motions of the apical domains are best appreciated in an end view movie of the whole ring (Movie S2).

Considering the individual rings of the Rd states, a similar set of conformational changes is observed, but in smaller steps, presumably due to the inhibitory effect of negative cooperativity between the two ATP-occupied rings. The E386-K80 contact is not formed in any of the Rd rings because the equatorial ring is slightly expanded in all of them. There is a more complex interplay between E255 and E257 at the end of helix I and K242 and K245 at the end of helix H (Figure 4B, Rd1-5). E257 is a highly conserved residue and has been implicated in substrate stimulation of ATP hydrolysis (Danziger et al., 2006; Brocchieri and Karlin, 2000). In the sequence of apical domain movements, the salt-bridge contact slips from 255–245 to 255/257–245 and then to 255/257–245/242, suggesting a "click stop" mechanism. Finally, all of these salt-bridge contacts are broken, and the apical domains detach from each other to form the Rd-open state (Figure 4B). These contacts determine the switch from a continuous belt of hydrophobic binding sites lining the open cavity to an expanded set of disconnected sites (see Movies S3 and S4).

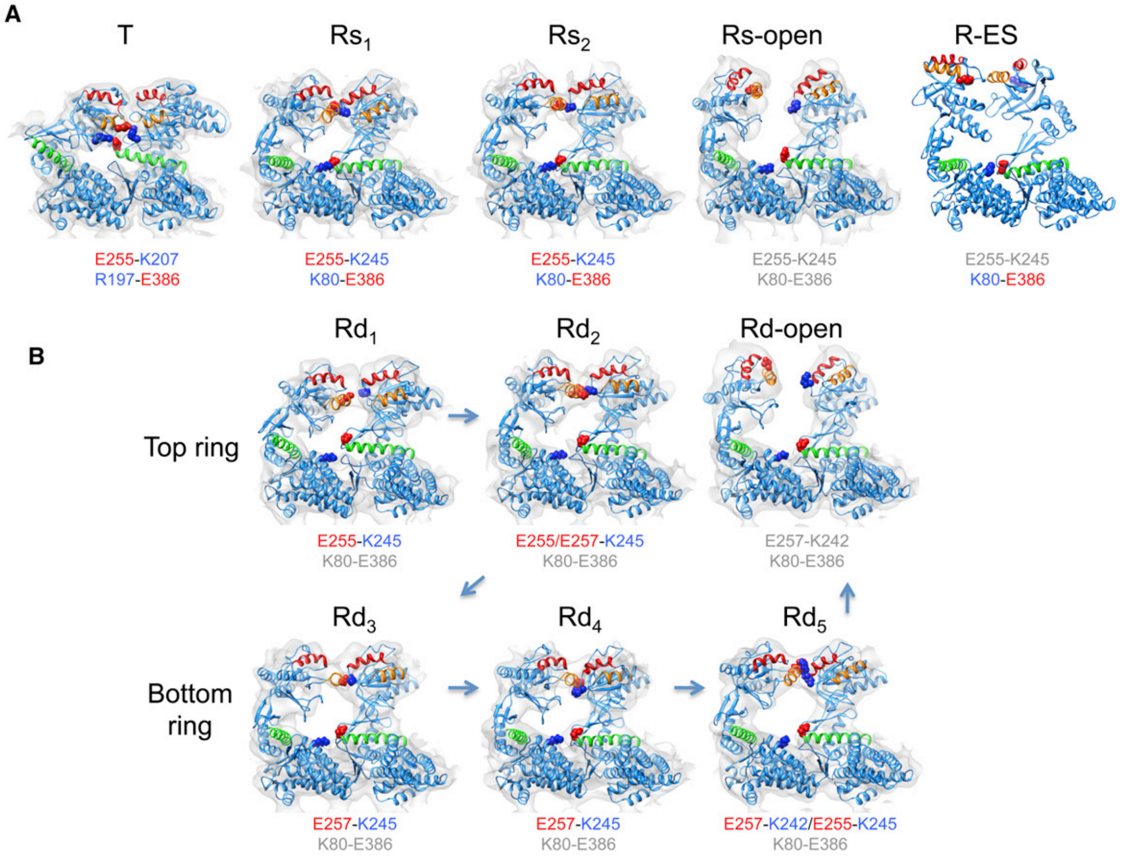

Figure 4. Subunit Conformations and Intersubunit Salt Bridges in GroEL Rings

Two adjacent subunits are shown as seen from inside the cavity, with EM density in white, helix H in red, helix I in orange, and helix M in green. Charged residues involved in intersubunit contacts are shown as spheres, with negatively charged residues in red, and positive ones in blue, and the contacts are listed for each ring. (A) GroEL-ATP_7 structures. For comparison, the crystal conformation bound to GroES (PDB ID 1SVT) is also shown (R-ES). (B) GroEL-ATP_{14} structures. The arrows in the Rd series indicate the sequence of states inferred from the salt-bridge changes and the smallest movement in each step. The contact residues are listed in gray when the contact distance (defined in Table S2) is greater than 8 Å. See also Figures S4 and S5, Movies S1, S2, S3, and S4, and Tables S1 and S2.

The transition to the R-open state is the main point at which misfolded substrates, if already bound to multiple adjacent apical domains, could be expanded in a forced unfolding step.

Structural Basis for Negative Inter-ring Cooperativity

GroEL is a member of the group 1 chaperonin subfamily, found in bacteria, mitochondria, and chloroplasts. In this subfamily, the two seven-subunit rings are staggered, so that each subunit contacts two others in the opposite ring. These contacts transmit the signals for negative cooperativity between the rings. In GroEL-ATP rings, the equatorial domains pivot, relative to the unliganded state, about the inter-ring contact containing the salt bridge R452-E461. Pivoting about this contact lengthens the other inter-ring contact, A109-A109, which is directly linked to the ATP γ-phosphate by helix D and provides a route for communication between the two rings (Braig et al., 1994; Roseman et al., 1996). This route is altered in the presence of ATP, potentially explaining negative cooperativity between rings.

We are now able to describe these movements in more detail (Figure 5). Unexpectedly, the equatorial domain rotations result in rotation and expansion of the whole ring. The T-R transition can be described as two movements in the inter-ring interface. The first is a downward tilt of the equatorial domain centered on the R452/E461/V464 contact, and the second is an anticlockwise rotation of the whole ATP-bound ring relative to the empty ring. This second movement was not previously detected and causes the A109-A109 inter-ring contact to change from tilted to vertical, which is clearly seen in both the density and the fitted coordinates. The tilt and rotation lengthen the A109 contact by $\sim$2 Å. In the transition from Rs to Rd and especially to R-open states, the equatorial domains move radially outwards, lengthening both inter-ring contacts. The inter-ring contact distances, specified in Table S2, are plotted in Figure S5. Movies of the ring interface, seen in a central slice and from outside the complex for the Rs and Rd series, show how the equatorial domain tilts progressively lengthen the A109 contact and weaken the whole interface (Movies S5, S6, S7, and S8). Qualitatively similar but larger tilts are seen in the equatorial domains of group 2 chaperonins, which are less constrained by having 1:1 instead of 1:2 contacts across the ring interface (Clare et al., 2008; Huo et al., 2010).

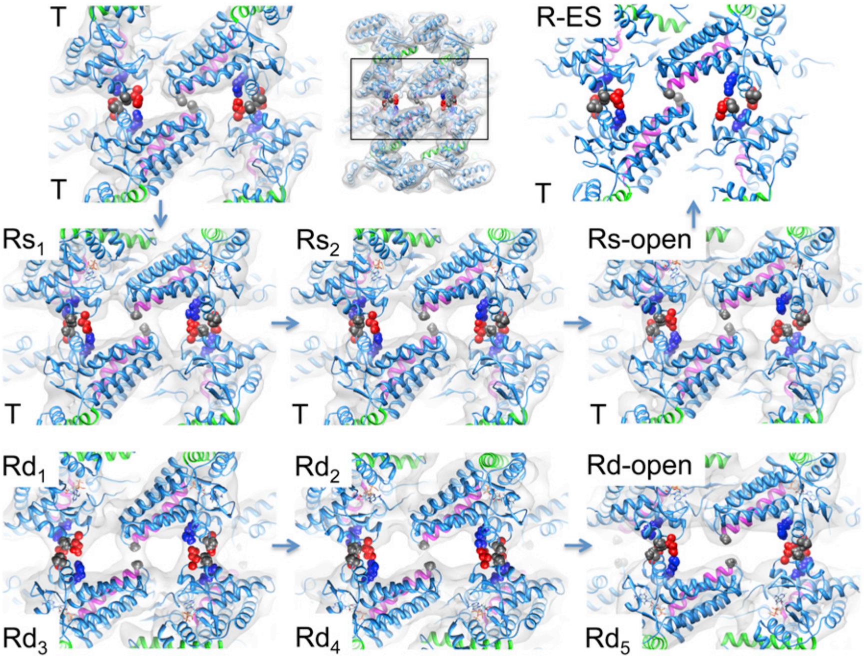

Figure 5. Inter-ring Interfaces in the GroEL Complexes

The salt-bridge contact involves E461 (red) and R452 (blue), along with the van der Waals contact V464-V464 (gray), seen in the left-hand contact in each panel. The contact at A109-A109 (gray, middle contact) is at the C-terminal end of helix D (magenta). D87 at the N terminus of helix D is involved in coordinating the ATP γ-phosphate. For the comparison with the GroES-bound state, we used the EM-derived model (PDB ID 2C7C) because the ring interface conformation in the crystal lattice is different from the one observed in solution (Ranson et al., 2006). The region viewed is indicated by the box in the overview (top, central panel). See also Figure S5, Movies S5, S6, S7, and S8, and Tables S1 and S2.

When ATP binds in the second ring, the equatorial domains recapitulate the movements seen in the first ring. This has the effect of increasing the distance between both contacts, with the interface at its weakest in the Rd-open:Rd5 complex, manifested as reduced or absent density for the A109 contact and very weak density at the R452/E461/V464 contact. The weak interfaces of the R:R states are consistent with previous observations of ring dissociation in ATP (Burston et al., 1996; Ranson et al., 2001, 2006).

Another unexpected finding is that the C termini, which are disordered in apo GroEL, are more ordered in all the ATP-bound rings (Figure S2). The cause and significance of this behavior are unknown, but it would seal the base of each ring, preventing even small substrates from crossing internally between rings.

DISCUSSION

The new observations of detachment and partial elevation of the apical domains in the presence of ATP, passing through Rs/Rd states to produce an R-open state, have major implications for binding and mechanical action of GroEL on folding substrates, as well as identifying the likely conformation for GroES docking. It was previously assumed that ATP-bound conformations of GroEL had low affinity for unfolded substrate. However a more recent study revealed that ATP binding is much faster than substrate binding (Tyagi et al., 2009), implying that, under physiological conditions, substrates are captured by a GroEL ring already occupied by ATP, here implied to be the Rs states.

In the apo GroEL structure, helices H and I demarcate a continuous collar of hydrophobic sites around the inner face of the GroEL ring (Figure 6A). The previous cryo-EM study (Ranson et al., 2001) suggested that the binding site is rotated into the intersubunit interface upon ATP binding, thus weakening substrate binding. However, our new data show that after ATP binding, adjacent apical domains are linked by intersubunit salt bridges between the ends of helices H and I, thus maintaining the continuous collar of binding sites facing the cavity, albeit with different orientations of the plane of the binding surface (Figure 6B). It seems possible that binding of a range of substrate conformations could be facilitated by the variety of orientations presented by the various R states.

The continuous collar of binding sites is important because substrate binding to three or more adjacent apical domains is essential for productive folding of stringent (strictly GroEL-GroES dependent) substrates (Farr et al., 2000). At least for some stringent substrates, the more internal part of the hydrophobic site, namely helix I and the underlying segment, provides the preferred binding site (Elad et al., 2007; Clare et al., 2009).

When the apical domains of the Rs2 state undergo a further elevation and move radially outwards, all the intersubunit contacts between apical domains are broken, producing the R-open state (Figure 6C). This movement could apply an extension force to a multivalently bound substrate, relieving kinetically trapped, misfolded states (Shtilerman et al., 1999; Lin et al., 2008). Notably, however, binding to an apo GroEL ring alone can accomplish such stretching (Lin and Rye, 2004). Nevertheless, following substrate protein binding by ATP-bound R states, as observed here, stretching could be mechanically exerted by the transition to the R-open state. Although the radial expansion and elevation break the continuous hydrophobic binding surface, the movements place the helix H/I grooves (which face into the cavity in all the preceding states) into an upward-facing orientation (R-open) in line with the GroES mobile loops, allowing GroES to be captured, whereas the stretched substrate protein remains bound to the separated hydrophobic surfaces. These views show how the binding surface is distorted, elevated, and finally occluded at the key points in the trajectory, and they are color-coded by hydrophobicity in Figure S6.

Although the GroES-binding sites in R-open are individually aligned 100° from their final orientations in the plane of the

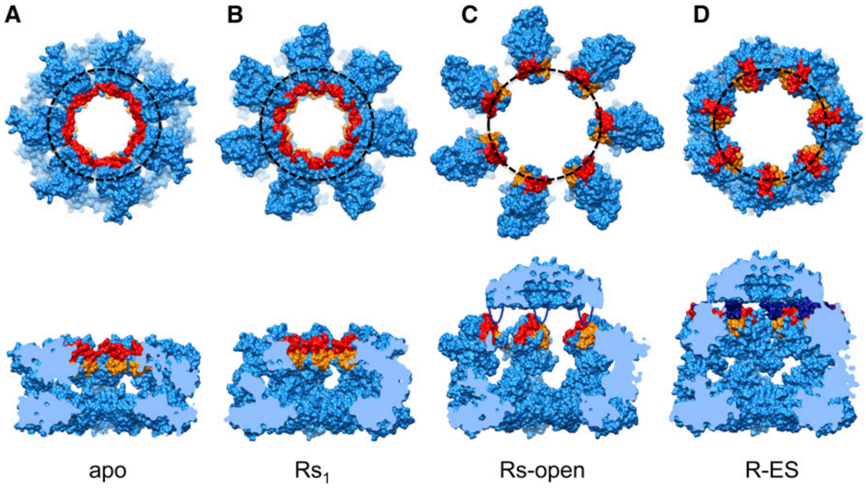

Figure 6. Footprint of GroES-Binding Sites on GroEL

The rings are seen in space-filling format from above and in cut-away side view in (A) apo, (B) Rs1, (C) Rs-open, and (D) R-ES (PDB ID 1SVT) rings. Helices H and I are in red and orange, respectively, and the binding residues on the mobile loops of GroES are shown in dark blue. The black dotted circle shows the radial distribution of GroES-binding sites. In the R-open state, the sites are at the same radius as in R-ES, but they are rotated by ~100°. GroES is schematically docked onto the R-open state to illustrate that the binding sites are readily accessible to the GroES mobile loops, unlike the situation in the Rs state. See also Figure S6.

GroEL-GroES-domed ring (compare Figures 6C and 6D), the mobile loops are sufficiently long and flexible to readily dock into the R-open ring from their unbound state (Hunt et al., 1996; Landry et al., 1996). Each loop could then twist 100° together with its bound apical domain to reach the final R-ES conformation. As indicated, GroES docking to the R-open state would explain why substrates do not escape before encapsulation. The major steps in the trajectory from apo GroEL to R-ES, via Rs1 and Rs-open, are shown as 3D cartoons in Figure 7. The indirect path from T to R-ES, in which the apical domains rotate sequentially in two orthogonal directions to reach the R-ES conformation, was not predicted from the known end-states and has major consequences for the mechanism of substrate encapsulation.

These major conformational steps are observed to have distinct patterns of intersubunit salt-bridge contacts that are visible in the EM density. The residues involved in the salt bridges formed upon ATP binding are reasonably well conserved in all GroEL sequences. Specifically, residues at the positions equivalent to *E. coli* residues 245 (K or R), 255 (E or D), and E257 involved in the interapical domain crosslink are conserved in bacterial and mitochondrial GroEL sequences, whereas K242 is not. In algal chloroplast GroEL sequences, all four charged residues are conserved, but in the higher plant chloroplast GroEL the β subunits have three out of the four (242, 255, and 257), and the α subunits only have one (257, the residue important in substrate stimulation of ATP hydrolysis; Danziger et al., 2006). However, the α subunits have an arginine at the position equivalent to 244 in the *E. coli* sequence, and this might be able to act as the salt-bridge partner to 255. Interestingly, K80, which forms the new equatorial-intermediate salt bridge to E386, is widely conserved in GroEL sequences except for the mitochondrial ones. These findings are supported by the analysis of a GroEL consensus sequence (Brocchieri and Karlin, 2000).

Previous mutational and biophysical studies provide additional support for the interpretation of movements presented here. In apo GroEL, there is an intrasubunit, equatorial-to-apical salt bridge D83-K327. The Cα separation between these residues goes from 8 Å in apo GroEL to 12.4 Å in Rs1 to 15.7 Å in Rs-open and finally to 36 Å in R-ES. Murai et al. (1996) showed that substituting D83 and K327 with cysteine (in a cysteineless, fully functional mutant version of GroEL) and oxidatively crosslinking the cysteines to lock down the apical domains blocked both GroES binding and ATP turnover. Conversely, in an independent experiment, the ability of A384C and S509C to oxidatively crosslink only in the presence of ATP confirms the large rotation of the intermediate domain characteristic of ATP-bound GroEL (see Figure 1 in Nojima and Yoshida, 2009).

A previous functional study provides strong support for an R-open-GroES intermediate. In the presence of bound substrate, the GroEL mutation C138W near the equatorial-intermediate domain hinge region produces a complex with both substrate protein and GroES bound, and this complex is arrested in ATPase and folding activities (Kawata et al., 1999; Miyazaki et al., 2002). This stalled state, formed at 25°C, is partly unblocked by warming to 37°, allowing folding to the native state to proceed. These findings fit extremely well with our proposal that Rs-open is an intermediate state that can simultaneously bind substrate and GroES. Our R-open structures provide a view of this intermediate conformation.

The sequence of ATP-directed GroEL states presented here is chosen to give the smallest domain movements in each step. The plausibility of these as distinct intermediates along a trajectory is supported by time-dependent fluorescence studies, which identify at least two distinct ATP-bound states that precede GroES binding (Taniguchi et al., 2004; Cliff et al., 2006). Notably, the states observed here by cryo-EM were all productive of the GroES-bound R-ES end-state: when GroES was added to D398A GroEL after ATP, we found by negative-stain EM that all available complexes bound GroES just as extensively as when GroES was added before ATP.

The energy driving the movements charted here must be derived from ATP binding. In previous studies, the energy of ADP binding alone was measured at ~45 kcal/mol of rings, whereas subsequent binding of an aluminum fluoride complex (simulating the γ-phosphate, albeit to an ADP/GroES-bound

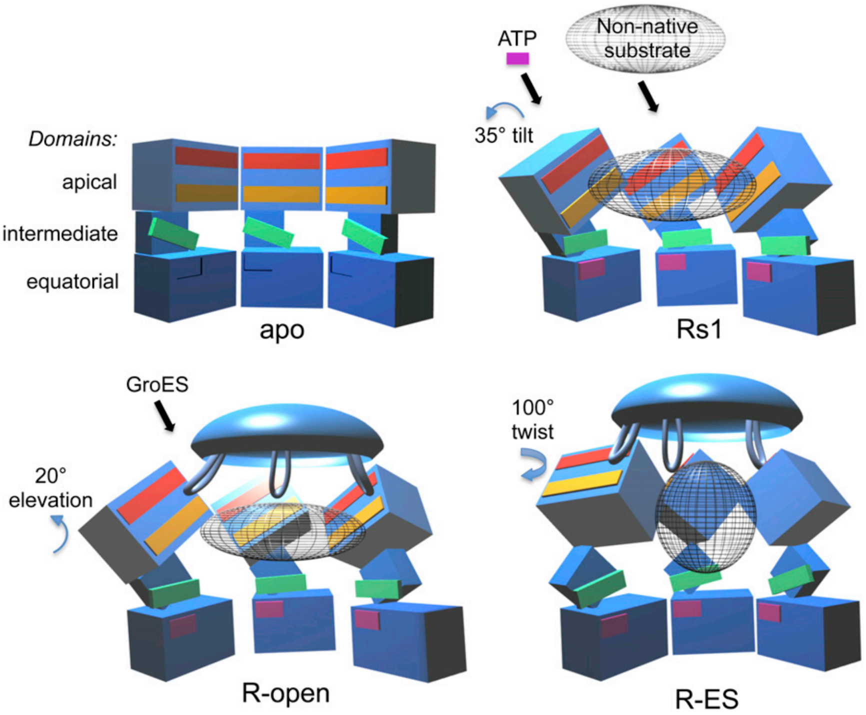

Figure 7. Cartoon of Domain Movements with Helices H, I, and M Highlighted in Apo, Rs, R-open, and R-ES States

Helices H (red), I (orange), and M (green) are highlighted. The folding substrate polypeptide is shown in gray mesh. GroES is shown docked onto the Rs-open state as in Figure 6C. The R-open apical domains have undergone about 70% of their elevation from apo to R-ES. This figure was generated in Blender 3D.

complex) was another ~45 kcal/mol of ring (Chaudhry et al., 2003). Thus it seems that there is ample energy supplied to allow the formation and breakage of salt bridges along the trajectory from the T state to R-open.

Conclusions: Implications for Chaperonin-Assisted Protein Folding

Important implications of this work for the mechanism of chaperonin action stem from the observation by cryo-EM of a set of distinct intermediate states formed in response to ATP binding that likely lie along a trajectory of movement. A key finding is that this trajectory, from an unliganded ring (T state) to the domed GroES/ATP-bound (R-ES state), is not direct. It seems to be comprised of two main phases. First, upon binding ATP, the GroEL apical domains tilt together with the intermediate domains (forming Rs1), begin to elevate by themselves (forming Rs2), and then detach from each other, expand radially, and elevate further (forming Rs-open). Substrate protein binds more slowly than ATP and thus in vivo is likely to bind to the tilting and elevating apical domains. During the expansion to Rs-open, the multivalently bound substrate must be exposed to a stretching force that could unfold trapped, misfolded conformations. This first phase ends with the R-open state. R-open holds bound polypeptide via hydrophobic contacts with at least helix I and the underlying segment, which still face the cavity, while simultaneously presenting binding sites for the GroES mobile loops at the correct positions for GroES docking. There is ample flexibility in the GroES loops to dock onto this conformation (Figures 6 and 7), and thus a ternary complex is formed from which captured, still-bound polypeptide cannot escape.

In the second phase of movement, following GroES docking, the further elevation of the apical domains and massive 100° clockwise twist provide the power stroke of chaperonin movement, which ejects the substrate protein from its binding sites by peeling away the remaining hydrophobic sites on helix I and the underlying segment. After this step, the released substrate would be free to collapse and fold inside the final, hydrophilic folding chamber.

The time course of the movements, based on observed rates of binding, would be rapid ATP binding (100 s^{-1}), followed by substrate binding and slower rearrangement (~5 s^{-1}), and then GroES docking (1–2 s^{-1}) (Taniguchi et al., 2004; Cliff et al., 2006; Tyagi et al., 2009). Therefore, our observed trajectory likely elucidates the encapsulation mechanism and indicates potentially where substrate protein can undergo an ATP-directed stretching action that would release it from misfolded states, as well as when it is ejected from the apical domains, followed by collapse and folding in the 70 Å long hydrophilic chamber. It provides a plausible mechanism by which the chaperonin releases proteins from kinetic traps and places them under conditions that favor productive protein folding to the native state without any stereospecificity and without the possibility of aggregation.

EXPERIMENTAL PROCEDURES

Protein Expression and Purification

GroEL expression and purification were done as previously described (Weissman et al., 1995; Rye et al., 1997).

Sample Preparation and EM

For the cryo-EM, 2.5 μl of 4 mg/ml GroEL in 50 mM Tris-HCl (pH 7.4), 50 mM KCl, and 10 mM $MgCl_2$ was applied to holey carbon-coated C-flat grids (r2/2, Protochips Inc., USA) followed by 0.5 μl of 400 or 800 μM ATP (ATP was added to the grids once they were mounted in the vitrobot). The grids were rendered hydrophilic with a Fischione plasma cleaner for 20 s in a specially adapted stage, then immediately used for sample preparation in a vitrobot (FEI, Netherlands) at 25°C and 100% humidity, blotted for 2–3 s, and rapidly plunged into liquid ethane maintained at −180°C. For GroEL-ATP complexes, a technical problem also encountered with GroEL-substrate complexes (Elad et al., 2007) reduced the number of side views to only 5%–10% of the total. In order to collect a very large data set, low-dose images were recorded with the automatic data acquisition system LEGINON on a Tecnai F20 electron microscope

operated at 120 kV and equipped with a Gatan cold stage (Gatan, USA) and recorded on a Gatan 4k CCD camera (Gatan, UK) at a magnification on the CCD camera of 148,500 with 0.7–3.5 μm underfocus. Approximately 6,000 frames were collected in multiple sessions.

Image Processing

The CCD cryo-EM images were 2 × 2 binned to 2.02 Å/pixel. The contrast transfer function (CTF) for each CCD image was determined with CTFFIND (Mindell and Grigorieff, 2003). Initially a small subset of particles was selected (side views only) using XIMDISP (Smith, 1999) and extracted into 256 by 256 boxes. The CTF correction was done in SPIDER (Frank et al., 1996), and after CTF correction, the boxes were cropped to 192 by 192. The images were filtered, normalized, and aligned to a filtered side-view projection of the apo GroEL crystal structure (Protein Data Bank [PDB] ID 1OEL; Braig et al., 1995). The total sum of the aligned images was then calculated and used as a reference template to automatically pick side views from all the CCD frames using FindEM (Roseman, 2004). The automatically picked particles were then extracted into 256 by 256 boxes, CTF corrected, and cropped as before. The boxed particles were band-pass filtered between 175 and 4 Å and then normalized. Images were initially centered using a projection of a filtered crystal structure from the side and classified by MSA in IMAGIC (van Heel et al., 1996) into classes containing approximately 6–10 images per class. Class averages that did not clearly represent GroEL side views were selected, and the images in these classes were removed from the data set. Following this, the remaining 60,000 images were aligned to reprojections from a reconstruction of unliganded GroEL. These aligned images were then classified using MSA into 10 subgroups in which only eigenimages describing conformational variation within rings and misalignment between particles were used (Figure S1). Based on these subpopulations, the images were partitioned into GroEL-ATP$_7$, GroEL-ATP$_{14}$, and "junk" (images not containing GroEL or with an obviously incorrect alignment). From this point, the 20,000 GroEL-ATP$_7$ images and 30,000 GroEL-ATP$_{14}$ images were treated as separate data sets.

For both data sets, the eigenimages were again calculated, and the ones reporting on variations in conformation were used to subdivide them into four subgroups. From these, two subgroups were selected for each data set, and reconstructions calculated. Alignment to reprojections of all these reconstructions was then used to refine the separation (Elad et al., 2007, 2008). This procedure was iterated until the movement of images between the two subgroups stabilized. Eigenimages calculated for both subpopulations in both data sets revealed that one subpopulation in each data set still had a significant conformational variation. Therefore, this information was used to split that subpopulation into two further subgroups from which reconstructions were calculated. This gave three distinct subgroups for each of the GroEL-ATP$_7$ and GroEL-ATP$_{14}$ data sets, which were then used as reference structures for competitive alignment. This procedure was iterated until the separation was reasonably stable, giving 5,500 images in each of the three GroEL-ATP$_7$ classes (Rs1, Rs2, and Rs-open) and 15,000, 6,500, and 6,500 images, respectively, in the three GroEL-ATP$_{14}$ classes (Rd5:Rd-open, Rd2:Rd4, and Rd1:Rd3). However, if the separation was left to run (up to iteration 16), images from Rs-open would slowly move to Rs2, causing the apical domains in Rs2 to expand and lose density. Therefore, to check the validity of the separations of Rs-open and Rs2 at iterations 9 and 16, asymmetric reconstructions were calculated. This test revealed that the apical domains at iteration 9 were in a similar position to that of the 7-fold maps, whereas at iteration 16, there was a lot of variation in apical domain position. Class Rs1 remained the same independent of the iteration number. This suggested that images of the Rs-open state were being incorrectly assigned to Rs2. A similar test was done with the GroEL-ATP$_{14}$ data set.

The images for each of the six ATP conformations were then split into separate data sets after iteration 9, and four further rounds of alignment and reconstruction were done. The angular distribution of the reference images (side views) was 80°–100° to the symmetry axis, over the asymmetric unit covering 0°–51.4° around the symmetry axis, with a step size of 1° for the final four rounds of alignment. Each of the six GroEL-ATP data sets and the apo data set were split into two halves and reconstructions generated, which were then soft edge masked and used to calculate the Fourier shell correlation (FSC). The resolution for each data set was estimated using the 0.5 criterion as 7 Å for apo GroEL and 8–9 Å for the GroEL-ATP complexes. The final reconstructions were filtered between 20–8.5 Å to correct for the over-representation of low-frequency information and to remove high-frequency noise. The low-frequency components were reduced to 10% of their original amplitudes.

Atomic Structure Fitting and Refinement

Starting from the apo rings of the GroEL-ATP$_7$ maps (the three T rings), the crystal structure of the full heptameric ring of apo GroEL (PDB ID 1OEL; Braig et al., 1995) was rigidly docked using Chimera (Pettersen et al., 2004), showing a good fit (cross-correlation coefficients: Rs1 = 0.616, Rs2 = 0.620, Rs-open = 0.633). Next, flexible fitting was applied with Flex-EM (Topf et al., 2008) to the ATP-bound rings from all six maps: Rs1, Rs2, and Rs-open in the GroEL-ATP$_7$ maps, and Rd1-5 and Rd-open in the GroEL-ATP$_{14}$ maps. Two crystal structures were used as starting points—apo GroEL$_{14}$ (PDB ID 1OEL) and GroEL$_{14}$-GroES$_7$ (PDB ID 1SVT; Chaudhry et al., 2004). Any mutations in the crystal structures used were rebuilt as wild-type using Chimera (Goddard et al., 2007), with the exception of the D398A mutation.

A flowchart of the fitting is shown in Figure S3. First, each map was box-segmented around one subunit. A single GroEL subunit was then refined in each segmented map. In some cases, additional manual intervention (performed in Chimera) was needed to complete the fit, followed by conjugate-gradient minimization of the physicochemical properties to reconnect the chain using MODELER (Sali and Blundell, 1993). In the flexible fitting procedure, the models were refined by optimizing the positions of nonhydrogen atoms with respect to their cross-correlation with the map, as well as to the stereochemical properties and nonbonded interactions, using simulated annealing molecular dynamics. The optimization was performed by defining groups of atoms that were treated as rigid bodies (e.g., domains), whereas the atoms in the hinge regions connecting the rigid bodies were treated individually.

Once a single subunit was fitted, the ring was rebuilt using C7 symmetry. For all the ATP-bound rings except Rs-open, the lateral β sheet contacts (residues 2–7 and 517–524 in one subunit and 37–49 in the neighboring subunit) were superimposed from 1OEL at the end of each fit to ensure that the correct intersubunit contact was constructed. Finally, to get a better description of the changes in nonbonded interactions between the different conformations (in particular intersubunit and interdomain interactions), the side chains of each of the six 14-mer models (which mostly were kept rigid within rigid bodies during the refinement) were energy minimized. The minimization was performed using NAMD2.6 (Phillips et al., 2005) with the CHARMM27 force field (Foloppe and MacKerell, 2000).

The specific procedures for each of the nine ATP-bound rings are described in the Extended Experimental Procedures. The final cross-correlation scores for the full double-ring fits into each map are as follows: Rs1:T = 0.657, Rs2:T = 0.651, Rs-open:T = 0.664, Rd1:Rd3 = 0.681, Rd2:Rd4 = 0.671, and Rd-open:Rd5 = 0.728.

Quantitative Analysis of the Fits

The angles, axes, and translations between the same domains in the different GroEL states were measured with MODELER (using an in-house script), by tracking the center of mass of each domain and the axis and angle of rotation needed for pairwise superposition of the domains (Topf et al., 2008). These measurements required prior alignment of all of the fits and crystal structures. The GroEL-ATP$_7$ fits, apo, and GroES-bound crystal structures were aligned by superposing the nucleotide-free rings. The GroEL-ATP$_{14}$ fits were aligned by rotating the structures around the z axis until the centers-of-mass of each subunit in the top ring were aligned. These measurements are shown in Table S1. The distances between interacting atoms in residues that form salt bridges are listed in Table S2.

ACCESSION NUMBERS

The cryo-EM maps of states apo, Rs1, Rs2, Rs-open, Rd1:Rd3, Rd2:Rd4, and Rd-open:Rd5 are deposited with Electron Microscopy Data Bank (EMDB) codes EMD-1997–2003, respectively. The PDB files fitted to the GroEL-ATP maps are deposited in the PDB as 4aaq (Rs1), 4aar (Rs2), 4aas (Rs-open), 4aau (Rd1:Rd3), 4ab2 (Rd2:Rd4), and 4ab3 (Rd-open:Rd5).

SUPPLEMENTAL INFORMATION

Supplemental Information includes Extended Experimental Procedures, six figures, two tables, and eight movies and can be found with this article online at doi:10.1016/j.cell.2012.02.047.

ACKNOWLEDGMENTS

We thank Richard Westlake, Dave Houldershaw, and Luchun Wang for computing and EM support; Wayne Fenton and Elena Orlova for discussion; and Helene Launay for help with the initial part of data analysis. We also thank Bridget Carragher, Clint Potter, Anchi Cheng, and the Leginon team for their help and access to the NRAMM facility at the Scripps Research Institute. This work was carried out at the National Resource for Automated Molecular Microscopy, supported by the National Institutes of Health though the National Center for Research Resources P41 program (RR017573) and at Crystallography, Department of Biological Sciences and ISMB, Birkbeck College, supported by Wellcome Trust programme grants (070776 and 089050) to H.R.S. D.V. was supported by a BBSRC PhD studentship. M.T. is supported by an MRC Career Development Award (G0600084) and the Human Frontier Science Program (RGY0079/2009-C).

Received: July 20, 2011
Revised: October 24, 2011
Accepted: February 6, 2012
Published online: March 22, 2012

REFERENCES

Braig, K., Otwinowski, Z., Hegde, R., Boisvert, D.C., Joachimiak, A., Horwich, A.L., and Sigler, P.B. (1994). The crystal structure of the bacterial chaperonin GroEL at 2.8 A. Nature *371*, 578–586.

Braig, K., Adams, P.D., and Brünger, A.T. (1995). Conformational variability in the refined structure of the chaperonin GroEL at 2.8 A resolution. Nat. Struct. Biol. *2*, 1083–1094.

Brocchieri, L., and Karlin, S. (2000). Conservation among HSP60 sequences in relation to structure, function, and evolution. Protein Sci. *9*, 476–486.

Burston, S.G., Weissman, J.S., Farr, G.W., Fenton, W.A., and Horwich, A.L. (1996). Release of both native and non-native proteins from a cis-only GroEL ternary complex. Nature *383*, 96–99.

Chaudhry, C., Farr, G.W., Todd, M.J., Rye, H.S., Brunger, A.T., Adams, P.D., Horwich, A.L., and Sigler, P.B. (2003). Role of the gamma-phosphate of ATP in triggering protein folding by GroEL-GroES: function, structure and energetics. EMBO J. *22*, 4877–4887.

Chaudhry, C., Horwich, A.L., Brunger, A.T., and Adams, P.D. (2004). Exploring the structural dynamics of the E.coli chaperonin GroEL using translation-libration-screw crystallographic refinement of intermediate states. J. Mol. Biol. *342*, 229–245.

Clare, D.K., Stagg, S., Quispe, J., Farr, G.W., Horwich, A.L., and Saibil, H.R. (2008). Multiple states of a nucleotide-bound group 2 chaperonin. Structure *16*, 528–534.

Clare, D.K., Bakkes, P.J., van Heerikhuizen, H., van der Vies, S.M., and Saibil, H.R. (2009). Chaperonin complex with a newly folded protein encapsulated in the folding chamber. Nature *457*, 107–110.

Cliff, M.J., Kad, N.M., Hay, N., Lund, P.A., Webb, M.R., Burston, S.G., and Clarke, A.R. (1999). A kinetic analysis of the nucleotide-induced allosteric transitions of GroEL. J. Mol. Biol. *293*, 667–684.

Cliff, M.J., Limpkin, C., Cameron, A., Burston, S.G., and Clarke, A.R. (2006). Elucidation of steps in the capture of a protein substrate for efficient encapsulation by GroE. J. Biol. Chem. *281*, 21266–21275.

Danziger, O., Shimon, L., and Horovitz, A. (2006). Glu257 in GroEL is a sensor involved in coupling polypeptide substrate binding to stimulation of ATP hydrolysis. Protein Sci. *15*, 1270–1276.

Elad, N., Farr, G.W., Clare, D.K., Orlova, E.V., Horwich, A.L., and Saibil, H.R. (2007). Topologies of a substrate protein bound to the chaperonin GroEL. Mol. Cell *26*, 415–426.

Elad, N., Clare, D.K., Saibil, H.R., and Orlova, E.V. (2008). Detection and separation of heterogeneity in molecular complexes by statistical analysis of their two-dimensional projections. J. Struct. Biol. *162*, 108–120.

Farr, G.W., Furtak, K., Rowland, M.B., Ranson, N.A., Saibil, H.R., Kirchhausen, T., and Horwich, A.L. (2000). Multivalent binding of nonnative substrate proteins by the chaperonin GroEL. Cell *100*, 561–573.

Fenton, W.A., Kashi, Y., Furtak, K., and Horwich, A.L. (1994). Residues in chaperonin GroEL required for polypeptide binding and release. Nature *371*, 614–619.

Foloppe, N., and MacKerell, A.D. (2000). All-atom empirical force field for nucleic acids: I. Parameter optimization based on small molecule and condensed phase macromolecular target data. J. Comput. Chem. *21*, 86–104.

Frank, J., Radermacher, M., Penczek, P., Zhu, J., Li, Y., Ladjadj, M., and Leith, A. (1996). SPIDER and WEB: processing and visualization of images in 3D electron microscopy and related fields. J. Struct. Biol. *116*, 190–199.

Goddard, T.D., Huang, C.C., and Ferrin, T.E. (2007). Visualizing density maps with UCSF Chimera. J. Struct. Biol. *157*, 281–287.

Hartl, F.U., and Hayer-Hartl, M. (2002). Molecular chaperones in the cytosol: from nascent chain to folded protein. Science *295*, 1852–1858.

Horovitz, A., Amir, A., Danziger, O., and Kafri, G. (2002). Phi value analysis of heterogeneity in pathways of allosteric transitions: Evidence for parallel pathways of ATP-induced conformational changes in a GroEL ring. Proc. Natl. Acad. Sci. USA *99*, 14095–14097.

Horwich, A.L., and Fenton, W.A. (2009). Chaperonin-mediated protein folding: using a central cavity to kinetically assist polypeptide chain folding. Q. Rev. Biophys. *42*, 83–116.

Hunt, J.F., Weaver, A.J., Landry, S.J., Gierasch, L., and Deisenhofer, J. (1996). The crystal structure of the GroES co-chaperonin at 2.8 A resolution. Nature *379*, 37–45.

Huo, Y., Hu, Z., Zhang, K., Wang, L., Zhai, Y., Zhou, Q., Lander, G., Zhu, J., He, Y., Pang, X., et al. (2010). Crystal structure of group II chaperonin in the open state. Structure *18*, 1270–1279.

Kawata, Y., Kawagoe, M., Hongo, K., Miyazaki, T., Higurashi, T., Mizobata, T., and Nagai, J. (1999). Functional communications between the apical and equatorial domains of GroEL through the intermediate domain. Biochemistry *38*, 15731–15740.

Landry, S.J., Taher, A., Georgopoulos, C., and van der Vies, S.M. (1996). Interplay of structure and disorder in cochaperonin mobile loops. Proc. Natl. Acad. Sci. USA *93*, 11622–11627.

Lin, Z., and Rye, H.S. (2004). Expansion and compression of a protein folding intermediate by GroEL. Mol. Cell *16*, 23–34.

Lin, Z., Madan, D., and Rye, H.S. (2008). GroEL stimulates protein folding through forced unfolding. Nat. Struct. Mol. Biol. *15*, 303–311.

Ma, J., Sigler, P.B., Xu, Z., and Karplus, M. (2000). A dynamic model for the allosteric mechanism of GroEL. J. Mol. Biol. *302*, 303–313.

Mayhew, M., da Silva, A.C., Martin, J., Erdjument-Bromage, H., Tempst, P., and Hartl, F.U. (1996). Protein folding in the central cavity of the GroEL-GroES chaperonin complex. Nature *379*, 420–426.

Mindell, J.A., and Grigorieff, N. (2003). Accurate determination of local defocus and specimen tilt in electron microscopy. J. Struct. Biol. *142*, 334–347.

Miyazaki, T., Yoshimi, T., Furutsu, Y., Hongo, K., Mizobata, T., Kanemori, M., and Kawata, Y. (2002). GroEL-substrate-GroES ternary complexes are an important transient intermediate of the chaperonin cycle. J. Biol. Chem. *277*, 50621–50628.

Motojima, F., Chaudhry, C., Fenton, W.A., Farr, G.W., and Horwich, A.L. (2004). Substrate polypeptide presents a load on the apical domains of the chaperonin GroEL. Proc. Natl. Acad. Sci. USA *101*, 15005–15012.

Murai, N., Makino, Y., and Yoshida, M. (1996). GroEL locked in a closed conformation by an interdomain cross-link can bind ATP and polypeptide but cannot process further reaction steps. J. Biol. Chem. *271*, 28229–28234.

Nojima, T., and Yoshida, M. (2009). Probing open conformation of GroEL rings by cross-linking reveals single and double open ring structures of GroEL in ADP and ATP. J. Biol. Chem. *284*, 22834–22839.

Orlova, E.V., and Saibil, H.R. (2010). Methods for three-dimensional reconstruction of heterogeneous assemblies. Methods Enzymol. *482*, 321–341.

Pettersen, E.F., Goddard, T.D., Huang, C.C., Couch, G.S., Greenblatt, D.M., Meng, E.C., and Ferrin, T.E. (2004). UCSF Chimera—a visualization system for exploratory research and analysis. J. Comput. Chem. *25*, 1605–1612.

Phillips, J.C., Braun, R., Wang, W., Gumbart, J., Tajkhorshid, E., Villa, E., Chipot, C., Skeel, R.D., Kalé, L., and Schulten, K. (2005). Scalable molecular dynamics with NAMD. J. Comput. Chem. *26*, 1781–1802.

Ranson, N.A., Farr, G.W., Roseman, A.M., Gowen, B., Fenton, W.A., Horwich, A.L., and Saibil, H.R. (2001). ATP-bound states of GroEL captured by cryo-electron microscopy. Cell *107*, 869–879.

Ranson, N.A., Clare, D.K., Farr, G.W., Houldershaw, D., Horwich, A.L., and Saibil, H.R. (2006). Allosteric signaling of ATP hydrolysis in GroEL-GroES complexes. Nat. Struct. Mol. Biol. *13*, 147–152.

Roseman, A.M. (2004). FindEM—a fast, efficient program for automatic selection of particles from electron micrographs. J. Struct. Biol. *145*, 91–99.

Roseman, A.M., Chen, S., White, H., Braig, K., and Saibil, H.R. (1996). The chaperonin ATPase cycle: mechanism of allosteric switching and movements of substrate-binding domains in GroEL. Cell *87*, 241–251.

Rye, H.S., Burston, S.G., Fenton, W.A., Beechem, J.M., Xu, Z., Sigler, P.B., and Horwich, A.L. (1997). Distinct actions of cis and trans ATP within the double ring of the chaperonin GroEL. Nature *388*, 792–798.

Rye, H.S., Roseman, A.M., Chen, S., Furtak, K., Fenton, W.A., Saibil, H.R., and Horwich, A.L. (1999). GroEL-GroES cycling: ATP and nonnative polypeptide direct alternation of folding-active rings. Cell *97*, 325–338.

Sali, A., and Blundell, T.L. (1993). Comparative protein modelling by satisfaction of spatial restraints. J. Mol. Biol. *234*, 779–815.

Shtilerman, M., Lorimer, G.H., and Englander, S.W. (1999). Chaperonin function: folding by forced unfolding. Science *284*, 822–825.

Smith, J.M. (1999). Ximdisp—A visualization tool to aid structure determination from electron microscope images. J. Struct. Biol. *125*, 223–228.

Suloway, C., Pulokas, J., Fellmann, D., Cheng, A., Guerra, F., Quispe, J., Stagg, S., Potter, C.S., and Carragher, B. (2005). Automated molecular microscopy: the new Leginon system. J. Struct. Biol. *151*, 41–60.

Taniguchi, M., Yoshimi, T., Hongo, K., Mizobata, T., and Kawata, Y. (2004). Stopped-flow fluorescence analysis of the conformational changes in the GroEL apical domain: relationships between movements in the apical domain and the quaternary structure of GroEL. J. Biol. Chem. *279*, 16368–16376.

Tehver, R., Chen, J., and Thirumalai, D. (2009). Allostery wiring diagrams in the transitions that drive the GroEL reaction cycle. J. Mol. Biol. *387*, 390–406.

Thirumalai, D., and Lorimer, G.H. (2001). Chaperonin-mediated protein folding. Annu. Rev. Biophys. Biomol. Struct. *30*, 245–269.

Topf, M., Lasker, K., Webb, B., Wolfson, H., Chiu, W., and Sali, A. (2008). Protein structure fitting and refinement guided by cryo-EM density. Structure *16*, 295–307.

Tyagi, N.K., Fenton, W.A., and Horwich, A.L. (2009). GroEL/GroES cycling: ATP binds to an open ring before substrate protein favoring protein binding and production of the native state. Proc. Natl. Acad. Sci. USA *106*, 20264–20269.

van Heel, M., Harauz, G., Orlova, E.V., Schmidt, R., and Schatz, M. (1996). A new generation of the IMAGIC image processing system. J. Struct. Biol. *116*, 17–24.

Weissman, J.S., Hohl, C.M., Kovalenko, O., Kashi, Y., Chen, S., Braig, K., Saibil, H.R., Fenton, W.A., and Horwich, A.L. (1995). Mechanism of GroEL action: productive release of polypeptide from a sequestered position under GroES. Cell *83*, 577–587.

Weissman, J.S., Rye, H.S., Fenton, W.A., Beechem, J.M., and Horwich, A.L. (1996). Characterization of the active intermediate of a GroEL-GroES-mediated protein folding reaction. Cell *84*, 481–490.

White, H.E., Chen, S., Roseman, A.M., Yifrach, O., Horovitz, A., and Saibil, H.R. (1997). Structural basis of allosteric changes in the GroEL mutant Arg197—>Ala. Nat. Struct. Biol. *4*, 690–694.

Xu, Z., Horwich, A.L., and Sigler, P.B. (1997). The crystal structure of the asymmetric GroEL-GroES-$(ADP)_7$ chaperonin complex. Nature *388*, 741–750.

Yang, Z., Májek, P., and Bahar, I. (2009). Allosteric transitions of supramolecular systems explored by network models: application to chaperonin GroEL. PLoS Comput. Biol. *5*, e1000360.

Yifrach, O., and Horovitz, A. (1995). Nested cooperativity in the ATPase activity of the oligomeric chaperonin GroEL. Biochemistry *34*, 5303–5308.

Yifrach, O., and Horovitz, A. (1998). Transient kinetic analysis of adenosine 5′-triphosphate binding-induced conformational changes in the allosteric chaperonin GroEL. Biochemistry *37*, 7083–7088.

Supplemental Information

EXTENDED EXPERIMENTAL PROCEDURES

Steps in Atomic Structure Fitting

(1) Rs1: A single subunit of 1OEL was flexibly fitted, first as two rigid bodies (the equatorial domain and the combined intermediate and apical domains) for ten iterations. Next, seven rigid bodies (the three domains, with helices K and L and both lateral β sheet contacts separate) were fitted for an additional ten iterations.

(2) Rs2: The final fit of Rs1was rigidly fitted into the single subunit map, and flexible fitting was first applied using six rigid bodies (the three domains, with the lateral β sheet contacts and helix I separate) for ten iterations. A further ten iterations of flexible fitting were applied, this time with helices K and L rather than helix I separate.

(3) Rd1: The Rs1 fit was rigidly fitted into Rd1, and the apical domain was manually moved by ~6 Å. Flexible fitting was then applied for three iterations using seven rigid bodies (the three domains with helices H, I, and lateral β sheet contacts separate).

(4) Rd3: The Rs1 structure was rigidly fitted, and flexible fitting was applied using three rigid bodies (the three domains) for ten iterations. The lateral β sheet contacts were then manually repositioned.

(5) Rd2 and Rd4: The bottom subunit of the Rd1 final fit was rigidly fitted into the top ring (chains A-G). Flexible fitting was applied using nine rigid bodies (the three domains with the lateral β sheet contacts and helices K, L, H, and I separate) for ten iterations. The same procedure as Rd2 was applied for Rd4, but starting with the final fit of Rs2.

(6) Rd-open and Rd5: For both rings, the intermediate and equatorial domains of 1SVT were combined with the manually moved apical domain from 1OEL. Attempts at fitting starting from 1svt or Rs2/Rd2 conformations produced clearly worse results for the apical domain. Flexible fitting was applied for ten iterations using all secondary structures as separate rigid bodies in the apical and intermediate domains (allowing more flexibility in the system to compensate for missing density). The equatorial domain remained rigid.

(7) Rs-open: The Rd-open structure was rigidly fitted into a single subunit, and flexible fitting was applied using five rigid bodies (the three domains with helices K and L separate) for ten iterations.

SUPPLEMENTAL REFERENCE

Kyte, J., and Doolittle, R.F. (1982). A simple method for displaying the hydropathic character of a protein. J. Mol. Biol. *157*, 105–132.

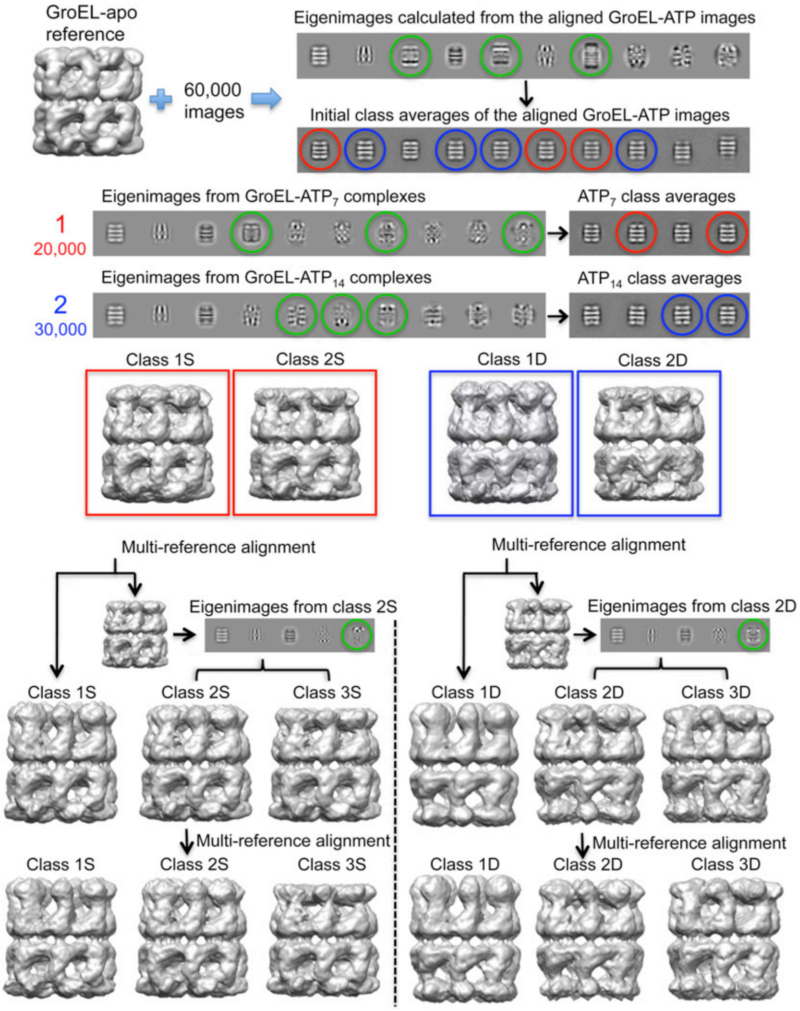

Figure S1. Schematic Outline of the Procedure Used to Sort the Data Set into Seven Distinct Structures, Related to Figure 2

The eigenimages of the whole, aligned data set indicate significant variations in the top and bottom of layers of the complexes, the locations of the apical domains. These eigenimages are circled in green. Class averages of this aligned data set show complexes with either one (red) or both (blue) apical domain layers extended relative to their appearance in apo GroEL. An initial rough sorting of the data into complexes with one (single, GroEL-ATP_7) or both (double, GroEL-ATP_{14}) rings occupied by ATP was based on the appearance of these classes. Eigenimages of the two subsets show more detailed features indicating variations in one or both apical domain layers. These eigenimages were used to further subdivide the two subsets, leading to the four structural classes whose 3D maps were used to generate reference projections for competitive alignment. Further eigenimage analysis led to one more subdivision of each set, yielding three single- and three double-ring ATP complexes.

Cell

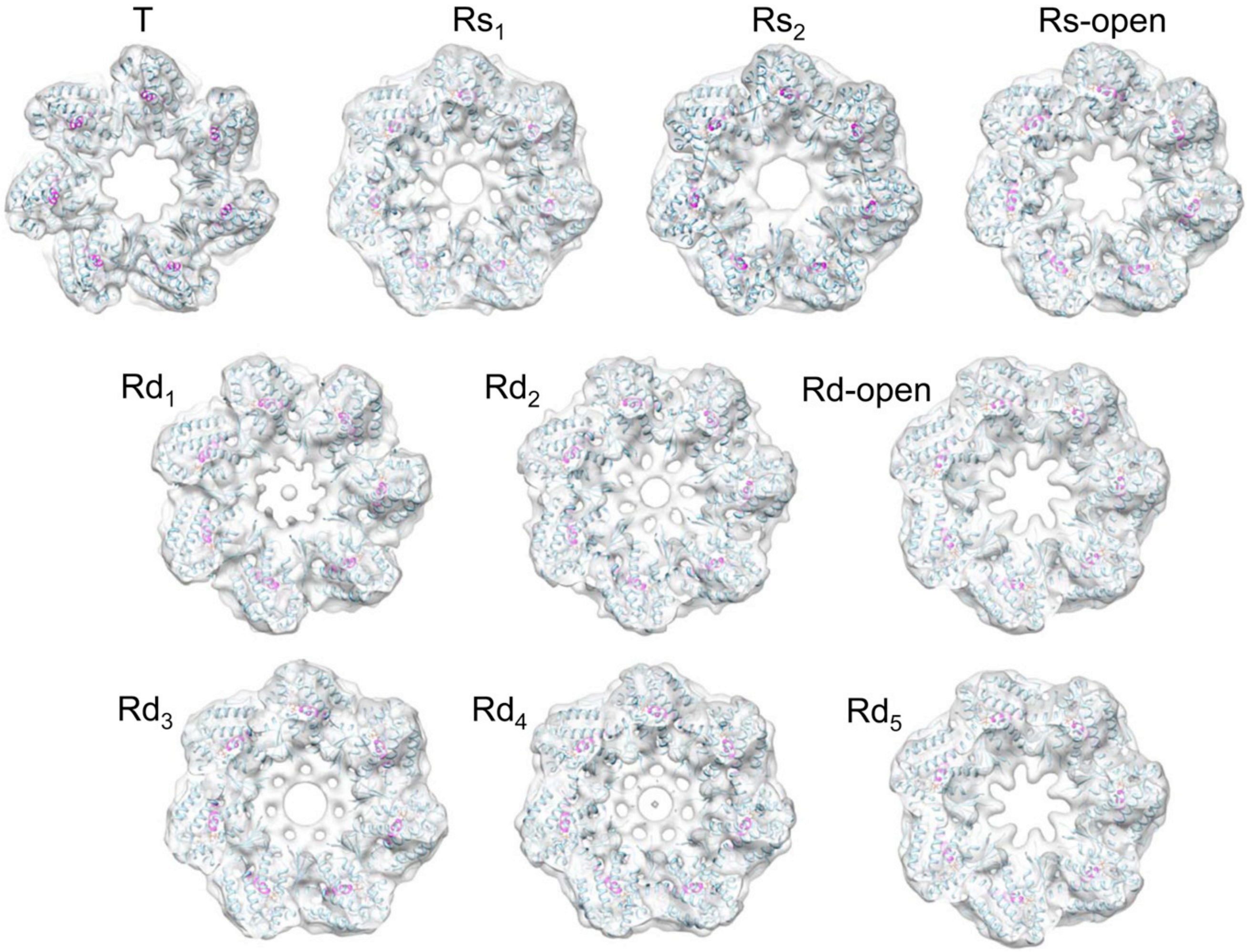

Figure S2. C-Terminal Densities, Related to Figure 2

Cross-sections of the maps and fits showing the additional density arising from the GroEL C termini, which are disordered in the T state and in the crystal structures. They become more ordered in ATP-bound rings.

Cell

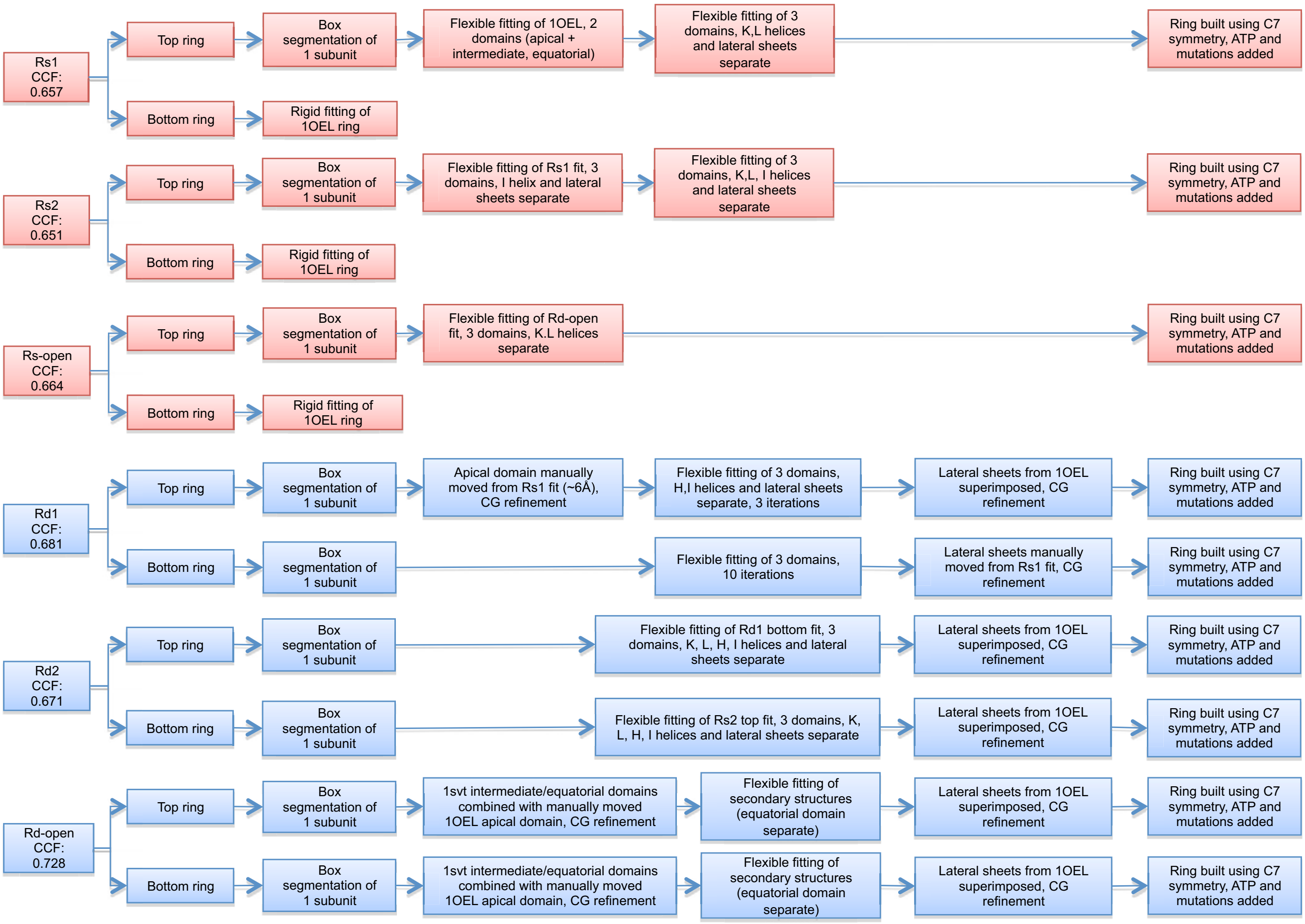

Figure S3. Flow Chart of Steps in the Flexible Fitting, Related to Figures 2 and 3

Lateral sheet superimposition in the equatorial domain contact was done by manual rigid fitting of the four strands from the 1OEL structure. All CG refinements were made without using map density. ATP was added by superimposing the equatorial domain from 1kp8. Mutations, rigid fitting, and segmentation done in Chimera, as were any manual changes.

ATP-bound ring of GroEL-ATP$_7$

Structures		Translation	Rotation	Rotation axis x	y	z
**Apical domains**						
Proposed trajectory						
Apo	Rs1	13.8	36.9	0.4	-0.6	0.7
Rs1	Rs2	3.0	8.9	1.0	0.1	-0.2
Rs2	Rs-open	10.3	17.7	0.1	0.4	-0.9
Rs-open	R-ES	7.6	95.6	-0.5	0.9	-0.2

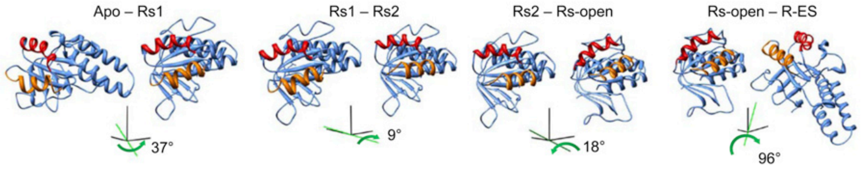

Figure S4. Apical Domain Rotations, Related to Figure 4

The main apical domain movements are tabulated, and the orientations of a pair of adjacent apical domains in the representative conformations are shown with reference frames to indicate the view orientation and the axis and angle of rotation at each step.

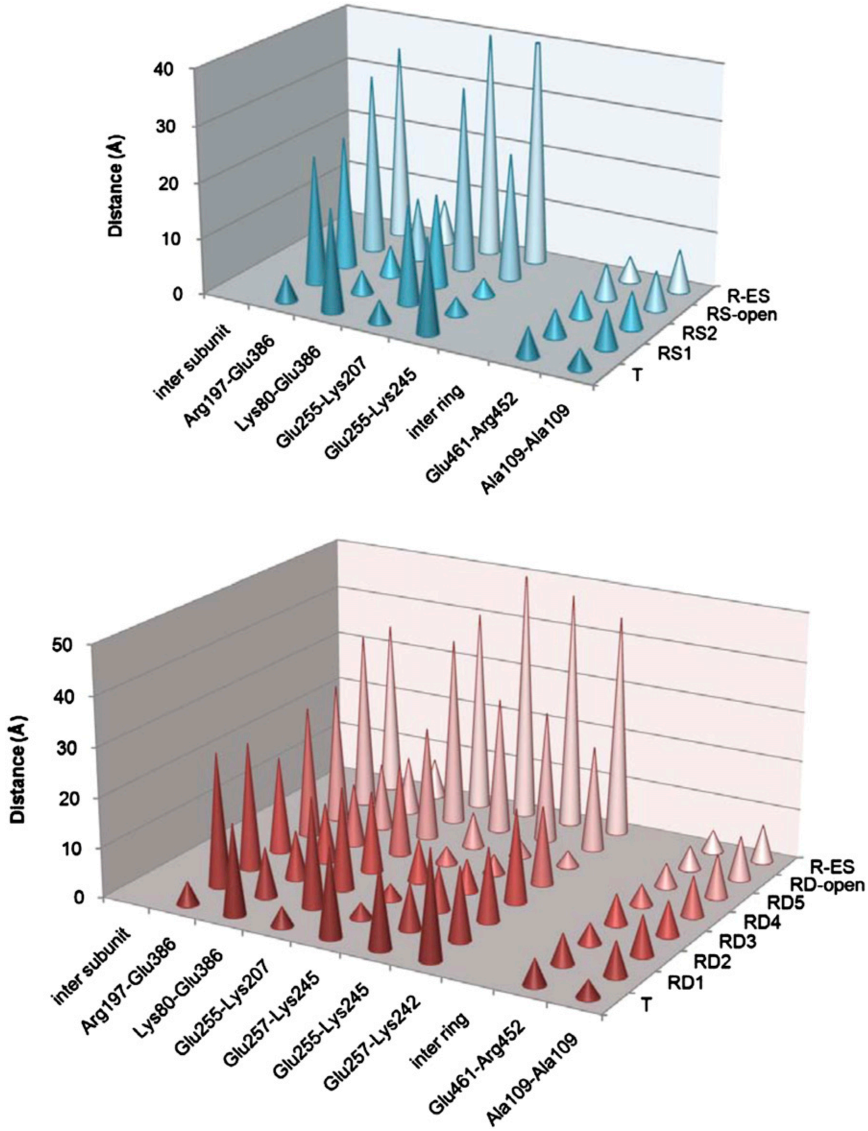

Figure S5. Plot of Intersubunit Contact Distances in the GroEL-ATP Complexes, Related to Figures 4 and 5

GroEL-ATP$_7$ (top) and GroEL-ATP$_{14}$ (bottom) complexes. The distances plotted are averaged over all seven subunits for each ring.

Cell

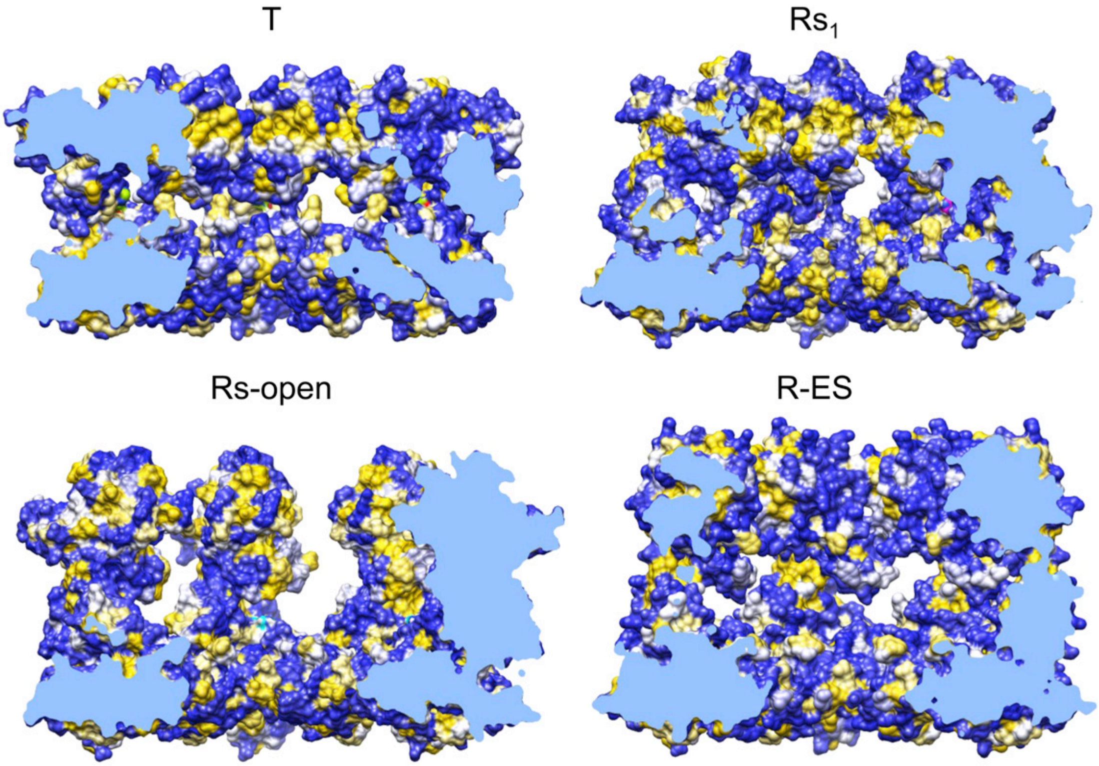

Figure S6. Surface Hydrophobicity Representations of Rings in the T, Rs1, Rs-open, and R-ES States, Related to Figure 6

The front half of each ring is cut away, with the cut surface shown in pale blue. Residue surfaces were colored using Chimera, based on the Kyte-Doolittle scale (Kyte and Doolittle, 1982), ranging from blue (most hydrophilic) through white to yellow (most hydrophobic). The continuous hydrophobic lining in the T state is distorted in Rs1, broken up in Rs-open, and occluded in R-ES.
